# Thermally Induced Phenomena in Amorphous Nifedipine: The Correlation Between the Structural Relaxation and Crystal Growth Kinetics

**DOI:** 10.3390/molecules30010175

**Published:** 2025-01-04

**Authors:** Roman Svoboda

**Affiliations:** Department of Physical Chemistry, Faculty of Chemical Technology, University of Pardubice, nam. Cs Legii 565, 532 10 Pardubice, Czech Republic; roman.svoboda@upce.cz

**Keywords:** amorphous nifedipine, structural relaxation, crystal growth, Raman microscopy, DSC

## Abstract

The particle size-dependent processes of structural relaxation and crystal growth in amorphous nifedipine were studied by means of non-isothermal differential scanning calorimetry (DSC) and Raman microscopy. The enthalpy relaxation was described in terms of the Tool–Narayanaswamy–Moynihan model, with the relaxation motions exhibiting the activation energy of 279 kJ·mol^−1^ for the temperature shift, but with a significantly higher value of ~500 kJ·mol^−1^ being obtained for the rapid transition from the glassy to the undercooled liquid state (the latter is in agreement with the activation energy of the viscous flow). This may suggest different types of relaxation kinetics manifesting during slow and rapid heating, with only a certain portion of the relaxation motions occurring that are dependent on the parameters of a given temperature range and time frame. The DSC-recorded crystallization was found to be complex, consisting of four sub-processes: primary crystal growth of α_p_ and β_p_ polymorphs, enantiotropic β_p_ → β_p_′ transformation, and β_p_/β_p_′ → α_p_ recrystallization. Overall, nifedipine was found to be prone to the rapid glass-crystal growth that occurs below the glass transition temperature; a tendency of low-temperature degradation of the amorphous phase markedly increased with decreasing particle size (the main reason being the increased number of surface and bulk micro-cracks and mechanically induced defects). The activation energies of the DSC-monitored crystallization processes varied in the 100–125 kJ·mol^−1^ range, which is in agreement with the microscopically measured activation energies of crystal growth. Considering the potential correlations between the structural relaxation and crystal growth processes interpreted within the Transition Zone Theory, a certain threshold in the complexity and magnitude of the cooperating regions (as determined from the structural relaxation) may exist, which can lead to a slow-down of the crystal growth if exceeded.

## 1. Introduction

Nifedipine (NIF), a calcium channel blocker, is a versatile medication used in various medical conditions. It is commonly employed in obstetrics as a tocolytic agent to delay preterm labor [1,2,3,4]. Additionally, nifedipine is utilized in the management of hypertensive disorders in pregnancy [5,6,7]. The drug’s mechanism of action involves blocking L-type calcium channels, which helps in relaxing smooth muscle and dilating blood vessels [8,9,10]. This vasodilatory effect is beneficial in conditions like hypertension, angina, and Raynaud’s disease [11,12,13]. Nifedipine has also been studied for its potential interactions with other medications, such as apatinib and ritonavir, which are important considerations for clinical use [14,15,16]. The drug has been investigated for its pharmacokinetics, including interactions with substances like omeprazole [17,18]. Studies have shown that nifedipine can be used safely and effectively in combination therapies [14,15]. The impact of nifedipine on various physiological parameters, such as blood pressure, cardiac function, and vascular responses, has been explored in [19,20,21]. It has been compared with other tocolytic agents like atosiban, showing varying outcomes in terms of perinatal mortality [4]. Additionally, nifedipine’s efficacy has been compared with other antihypertensive medications, highlighting its role in managing cardiovascular conditions [5].

Nifedipine’s wide-ranging applications in obstetrics, cardiology, and other medical specialties underscore its importance as a therapeutic agent, and its vasodilatory and smooth muscle relaxant properties make it a valuable tool in the management of conditions such as preterm labor and hypertension. However, due to its low water solubility (<5 μg·mL^−1^ [22]), the dissolution and bioavailability of NIF is very limited [23,24] unless enhanced via various formulations [23,24,25,26]. One of the easiest alternative ways of increasing bioavailability is the utilization of the drug in its amorphous form. However, the indisputable advantages of the amorphous drugs (enhanced bioavailability, uniformity of the amorphous state itself in comparison to the crystalline polymorphs, circumventing patent laws, etc.) are largely compensated by the disadvantage of the potential spontaneous uncontrolled degradation (recrystallization) of the amorphous state. Such formation of the crystalline phase in an amorphous drug is highly undesirable, as it can lead to unpredictable changes in the released dosage or even to the formation of potentially inactive/harmful polymorphs [27]. Therefore, it is of utmost importance to identify and describe the crystal growth mechanism and kinetics for every active pharmaceutical ingredient (API) that is intended to be used in amorphous form.

The crystal growth from amorphous NIF can result in the formation of a variety of polymorphic phases with differing melting temperatures *T_m_*: α_p_ (171 °C), β_p_ (recrystallizing at 70 °C into β′_p_ with *T_m_* = 164 °C), γ_p_ (recrystallizing at 61 °C into γ′ with *T_m_* = 137 °C), and δ_p_ (*T_m_* = 157 °C) [28,29,30,31,32]. Note that subscript “p” is used only within the framework of the present paper to avoid later confusion with certain physico-chemical quantities. Whereas the α_p_ polymorph is thermodynamically stable, the metastable β_p_ and γ_p_ polymorphs are kinetically favored and exhibit 3–4 orders of magnitude higher nucleation rates in the 30–110 °C temperature range [28]. The NIF crystal growth rates were extensively studied in [28,29,30,31,32,33,34,35,36,37,38,39], exploring various growth mechanisms in pure NIF glass, NIF/solvent, and NIF/polymer mixtures. In particular, the typical three crystal growth modes were identified in amorphous NIF: ordinary bulk growth, rapid diffusion-less glass-to-crystal (GC) sub-*T_g_* growth propagating primarily along the bulk micro-cracks (where *T_g_* is the glass transition temperature), and enhanced surface growth based on much higher surface mobility (compared to the bulk self-diffusion) [37]. The amorphous films formed by means of evaporation from solutions exhibited more than three orders of magnitude higher growth rates compared to the surface and bulk NIF growths [34]. The polymer additives then tend to stabilize the metastable polymorphic phases; they can also either accelerate or inhibit both the surface and bulk growths, and the presence of low polymer amounts can also lead to complete suppression of the enhanced surface and GC growth modes [30,32,36,37,38].

Whereas the microscopically determined NIF crystal growth rates are relatively well explored, the macroscopic manifestation of the crystal growth (monitored, e.g., by means of differential scanning calorimetry, DSC) was addressed only a few times [40,41,42,43]. More importantly, none of the macroscopic/calorimetric studies were performed systematically with respect to the major aspect driving the crystallization behavior, i.e., the particle size of the prepared amorphous powders or bulk pieces. Since it is well known that the low-molecular glasses (and APIs in particular) dominantly crystallize from the surface and that the presence of mechanical defects, sharp edges, and micro-cracks is essential for certain crystal growth modes [28,29,30,31,32,33,34,35,36,37,38], the aim of the present study is to thoroughly describe the changes in the crystallization and/or polymorphic kinetics that occur with decreasing size of the original amorphous powder grains. To cover the full range of pharmaceutically utilizable amorphous NIF powders, the particle sizes in the 50–1000 μm range (thus largely differing in their surface-to-volume ratio) will be investigated both calorimetrically and spectroscopically, and the corresponding kinetic predictions (considering both, storage and potential processing routes) will be introduced. In addition to the mathematico-physical description of the macroscopic crystal growth mechanisms, the kinetics of the structural relaxation movements (a process responsible for the glass transition phenomenon) will be measured and discussed in regard to its recently introduced mutual inter-relationship with the adjacent crystal growth, as discussed in [44,45,46,47,48,49].

## 2. Results

The present section will be structured as follows: First, the DSC data for the simple heating scans primarily designed to monitor the crystallization behavior will be introduced and described with respect to the potentially occurring polymorphic transitions (specifically, the cold crystallization, i.e., the crystallization occurring during the heating of the amorphous phase, will be studied). Second, the base quantification of the thermo-kinetic features (characteristic temperatures and enthalpy changes associated with the glass transition, crystallization, and melting) will be introduced. Third, the constant ratio (CR) and constant heating rate (CHR) cycles data obtained by DSC will be presented, demonstrating not only the structural relaxation behavior but also the conditions for the stability of the amorphous state of NIF. Fourth, the supplemental characterization data obtained by Raman and optical microscopies will be shown, revealing the nature of certain calorimetrically observed phenomena.

### 2.1. Base Calorimetric Measurements

**Figure 1:**
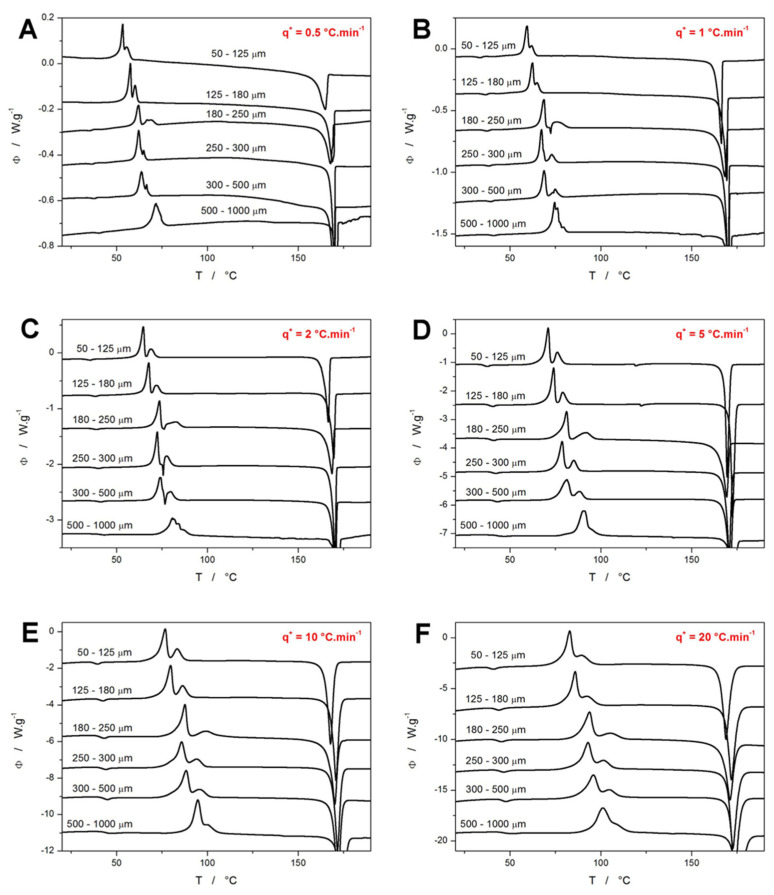
(**A**–**F**) DSC curves obtained for differently sized NIF powders at heating rates *q^+^* = 0.5–20 °C·min^−1^. Exothermic effects evolve in the upwards direction. The DSC curves were shifted along the Y axis to enhance the clarity.

**Figure 2:**
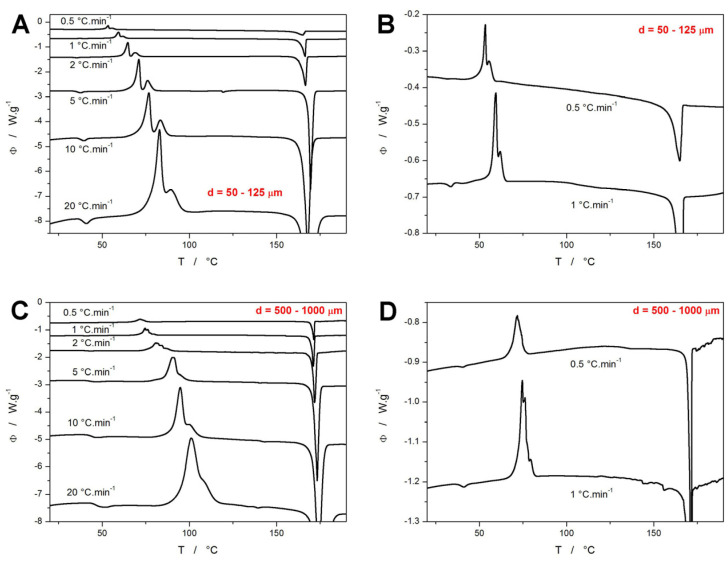
(**A**–**D**) DSC curves obtained for the 50–125 μm and 500–1000 μm NIF powders at heating rates *q^+^* = 0.5–20 °C·min^−1^. Graphs (**B**,**D**) are zoomed in on the crystallization peaks measured at the lowest *q^+^*. Exothermic effects evolve in the upwards direction. The DSC curves were shifted along the Y axis to enhance the clarity.

The base DSC data (the simple heating scans primarily performed to study the crystallization behavior of NIF) are shown in Figure 1. For better clarity, the data curves are sorted according to the applied heating rate *q^+^* and not the powder particle size; examples of the latter depiction are shown in Figure 2. As can be seen from the comparison of the DSC curves obtained for different powder sizes at the same *q^+^* (as shown in Figure 1), the crystallization peaks shift to lower *T* with increasing powder size, which is consistent with the idea of the crystal growth being primarily initiated from the surface-located defects (as is customary for low-molecular organic glasses [34,37]). The fact that this shift is not caused by thermal gradients (which could, theoretically, be larger in larger NIF grains) is confirmed by the invariability (or better, slight non-systematic variability) of the melting temperature *T_m_* and by the fact that similar temperature shifts in the crystallization peak are observed for both low and high *q^+^* (if the thermal gradients played any role, their influence would increase at high *q^+^*). The DSC crystallization signal itself is relatively complex, consisting of two main peaks, and under certain conditions (*q^+^* = 1–2 °C·min^−1^, middle-sized powders), a sharp endothermic peak occurs in between the two exothermic crystallization peaks. It should be noted that the endothermic process may also be (at least to some extent) present for other crystallization conditions, but its overlap with the prominent exothermic peaks may mask its manifestation.

The attribution of the thermal behavior features displayed in Figure 1 and Figure 2 to the particular polymorphic forms of NIF can be conducted in accordance with [28,29,30,31,32,34]. As evidenced by the onset of the melting temperature (the main endothermic melting peak), the achieved high-*T* crystallization state of NIF is represented by either the polymorph α_p_ (with the melting onset temperature *T_m_^ons^* = 171 °C) or the polymorph β′_p_ (*T_m_^ons^* = 164 °C); considering the often manifested broadness of the melting peak, the presence of both polymorphs is highly plausible in such cases. An occasional very weak endothermic melting signal near 137 °C suggests a minor presence of the γ′_p_ NIF polymorph in the case of the 500–1000 μm powders. On the other hand, a significantly stronger (but still weak) endothermic signal was recorded at ~120 °C for the fine NIF powders heated at 5 °C·min^−1^, which could not be attributed to any known polymorphic form or transition (given the very specific conditions for the occurrence of this signal, its potential cause being impurities is not very likely; technically, it might correspond to the melting of some unknown/rare polymorphic phase, the growth of which might be associated with potential impurities and the specific experimental conditions). The relatively strong endothermic peak near 75 °C then indicates the enantiotropic transition from the β_p_ to the β′_p_ polymorph. Based only on the calorimetric data, it is clear that the β_p_ polymorph needs to form during the first crystallization peak (manifesting within the complex crystallization signal) and that the formation of the α_p_ polymorph is not linked to any specific type of DSC peak complexity. Note that the crystallization signals differ in the degree of the overlap as well as in the relative magnitudes and asymmetries of the two exothermic peaks. As shown in Figure 2, for the fine powders, the shape of the crystallization signal was very consistent within each series of applied *q*^+^ (measurements performed for the powder with a given particle size). With increasing powder coarseness, irregularities occurred, as the same sample mass contained only a small number of NIF grains, and the specific states of each grain surface mattered more; this is particularly true at low *q^+^* when the preferential nucleation and/or crystal growth dominantly originates from the mechanical defects. The DSC data in Figure 2C,D are of particular interest, as several archetypal crystallization features can be recognized. Firstly, the gradual occurrence of the secondary high-T peak/shoulder is a consequence of the significant difference between the activation energies of the two corresponding sub-processes. Secondly, the “scatter” in the signal is caused by the simultaneous presence of only a few crystallization centers, when the consequent crystal growth within the given grain (or a large portion of its area) essentially manifests itself as a “separate” DSC peak. At very low *q*^+^, only the energetically most favorable crystallization centers initiate the growth (hence, the almost full overlap of all sub-signals). At high *q*^+^, the surplus energy activates many more crystallization centers (resulting in many more smaller peaks being overlapped), which together with the time/temperature-broadened DSC signal again results in a smooth overall envelope. Thus, only the middle-ranged *q*^+^ (1 and 2 °C·min^−1^) lead to the “scattered” signal.

### 2.2. Quantification of the Thermo-Kinetic Behavior

**Figure 3:**
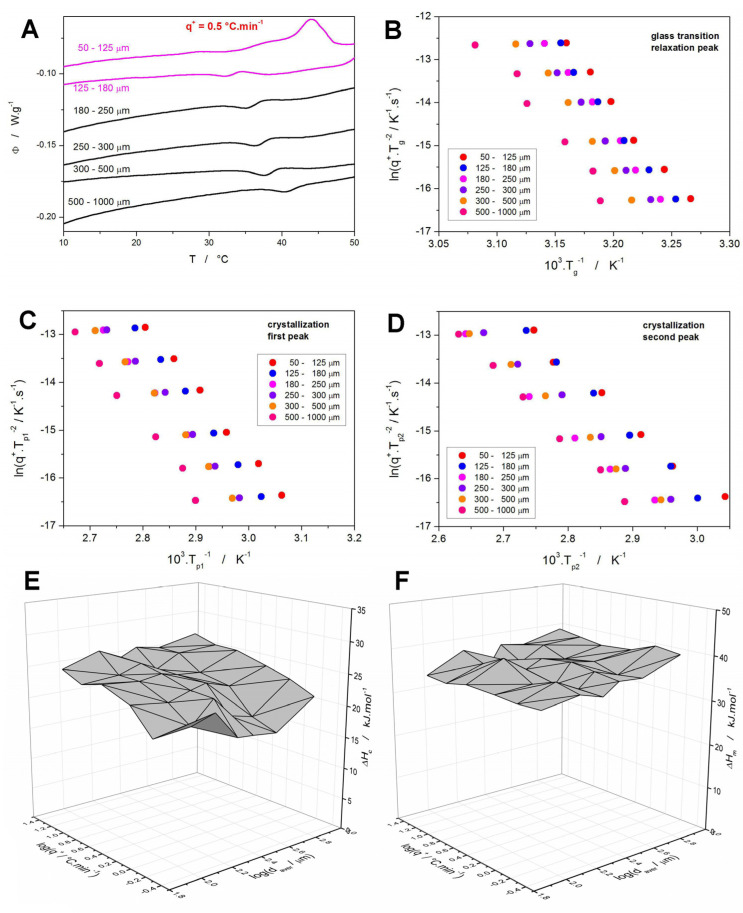
(**A**) DSC curves obtained for different NIF powders at *q^+^* = 0.5 °C·min^−1^—the graph is zoomed in on the glass transition region. The purple curves show the original measurements, during which nucleation and crystal growth occurred already during the idle period in the DSC autosampler. (**B**–**D**) Kissinger plots constructed for the *T_g_*, *T_p_*_1_, and *T_p_*_2_ quantities. (**E**,**F**) Crystallization and melting enthalpies obtained for the NIF powders in dependence on *q^+^* and *d_aver_*.

To quantify the above-described thermal behavior of the NIF powders with defined particle sizes, the characteristic temperatures and enthalpies were determined for the data depicted in Figure 1. In particular, the following quantities were monitored and are listed in the Supplementary Materials: the glass transition temperature determined as the half-height midpoint (*T_g_*), the glass temperature determined as the maximum of the relaxation peak (*T_g_^p^*), the temperature of the onset of the first crystallization peak (*T_ons_*), temperature of the maximum of the first crystallization peak (*T_p_*_1_), temperature of the maximum of the second crystallization peak (*T_p_*_2_), the temperature of the onset of the melting peak (*T_m_*), the temperature of the maximum of the melting peak (*T_m_^p^*), overall crystallization enthalpy (Δ*H_c_*), and melting enthalpy (Δ*H_m_*). Evolution of the selected quantities with *q^+^* and average powder particle size (*d_aver_* defined as the arithmetic mean of the maximum and minimum particle size of the given fraction) is shown in Figure 3, where the characteristic temperatures are plotted in the so-called Kissinger transformation, see Equation (1):

(1) $$ln\left(\frac{q^{+}}{T_{p}^{2}}\right)=-\frac{E}{RT_{p}}+const.$$

where *E* is the activation energy of the crystallization process, and *R* is the universal gas constant (8.314 J·mol^−1^·K^−1^).

The original trends in the depicted quantities revealed that for the fine powders, a complete series of *q^+^*-defined measurements exhibited curvatures inconsistent with constant nucleation/growth conditions being introduced for each measurement. In particular, the measurements performed at low *q^+^* (that were performed last in the series and thus, stayed at ~20 °C in the DSC autosampler for several hours) exhibited a significantly earlier crystallization onset. This was clearly a result of the additional nucleation and/or GC growth proceeding below *T_g_* during the DSC measurements queue, an example of such altered data is shown in Figure 3A. For the 125–180 μm powder, only a small exothermic crystallization signal overlaps with the glass transition effect (effectively decreasing the corresponding heat capacity change Δ*c_p_*, and manifesting as a very small exothermic peak in the vicinity of the glass transition endset); in the case of the 50–125 μm powder, the glass transition is barely identifiable due to the full overlap with an ongoing and further continuing crystal growth that evolves in the 33–47 °C range. Similarly, significantly less altered crystallization behavior was also observed in the case of these powders being heated at 1 °C·min^−1^ (the penultimate measurement in the series). Incidentally, this confirms the crucial influence of the presence of mechanical defects (micro-cracks, edges, etc.) for the macroscopic manifestation of the GC and enhanced surface growth modes in amorphous NIF. Note that these types of crystal growth are known [30,32,36,37,38] to originate from mechanical defects (micro-cracks are mainly reported in the literature). Since the crystallization process occurring just in the vicinity of *T_g_* (as depicted in Figure 3A) is a sign of these crystal growth modes being present (at least as an initiation of the macroscopic DSC recorded crystallization), their occurrence only in the case of the finest studied NIF powder with a vastly larger number of defects evidently interlinks these findings, and further supports the literature data. These alterations of the standard above-*T_g_* crystallization behavior were particularly well identifiable in the Kissinger graphs (Figure 3C,D), where the theoretical linearity of the ln(*q^+^*/*T_p_*^2^)−*T_p_*^−1^ dependences was largely violated for low *q^+^* of these two powder fractions. For this reason, the 50–125 μm and 125–180 μm powders were repeatedly prepared (including the new melt-quenches of new NIF glass ingots) specifically for each of these measurements (at 0.5 and 1 °C·min^−1^). The freshly prepared and immediately measured powders already exhibited unaltered crystallization (depicted in Figure 1 and Figure 2), as confirmed by the linearity of the Kissinger graph (Figure 3C) and based on the consistency of the shape of the crystallization peaks within the given *q^+^* series (e.g., Figure 2A).

Apart from the determination of the activation energy (which will be discussed in Section 4), the Kissinger plots can very effectively assess the temperature shifts in the monitored processes with various experimental conditions. This is particularly important in the case of the glass transition data, as shown in Figure 3B. Theoretically, *T_g_* could be expected to be invariant with powder particle size due to the manifestation of the relaxation motions only on a molecular level. This would imply that the shifts depicted in Figure 3B are related to the thermal gradients being present on the path from within the sample to the DSC sensor. However, such gradients would primarily manifest themselves as curvatures/shifts to higher *T* at higher *q^+^*, and their effect would cease for the data measured at *q^+^* = 0.5 °C·min^−1^ [50]. Since no such effect is observed in Figure 3B, the increase of *T_g_* with *d_aver_* is indeed a consequence of the relaxation processes being sped up by the presence of volume mechanical defects or (more probably) by the internal stress induced either by the melt-quench or by the gentle grinding of the amorphous NIF matter (note a similar manifestation in case of amorphous indomethacin [46]). Note that the Raman microscopy checks did not detect any traces of the crystalline phase in any of the as-prepared NIF powders, which rules out the potential influence of initial crystallinity (driven by particle size) on the shift of *T_g_*. Whereas the stress/quench-induced temperature shift of *T_g_* exhibits a monotonous trend with increasing *d_aver_*, the same cannot be stated about the crystallization peaks. The borderline powder sizes (50–125 μm, 125–180 μm, and 500–1000 μm) verify the fundamental idea of the crystal growth being accelerated in the presence of mechanical defects. However, the middle-sized powders, which do not have a very large amount of mechanical defects nor are they free of them, show variability and inconsistency with the expected monotonous increase in the crystallization characteristic temperatures (*T_ons_*, *T_p_*_1_, and *T_p_*_2_) with rising *d_aver_*. This unambiguously indicates that the intensity of the crystal growth induced by the presence of mechanical defects is comparable to that associated with the variable number of primary nuclei originating from the melt-quench process. Since, similar repeated preparations of the amorphous powders were recently performed for other low-molecular organic glasses (indomethacin [46], griseofulvin [51], nimesulide [52]), based on this experience, NIF seems to be most prone to the slight changes in the melt-quench procedure, which consequently affect its tendency to form the crystalline phase.

In addition to the characteristic temperatures, the enthalpies of crystallization and melting were determined, as shown in Figure 3E,F. Considering that the melting enthalpy is more-or-less constant (the slight decrease of Δ*H_m_* with decreasing *d_aver_* can be explained by a certain small portion of amorphous phase occupied by the defects being prevented from fully crystallizing) and that the NIF powders were fully amorphous when the DSC measurement was started (this was verified multiple times by means of Raman spectroscopy, as will be shown later), the significant decrease of Δ*H_c_* associated with decreasing *q^+^* must be related to the manifestation of Kirchhoff’s law, where the phase transition that occurs at lower *T* exhibits a smaller enthalpy change.

### 2.3. Structural Relaxation Measurements

**Figure 4:**
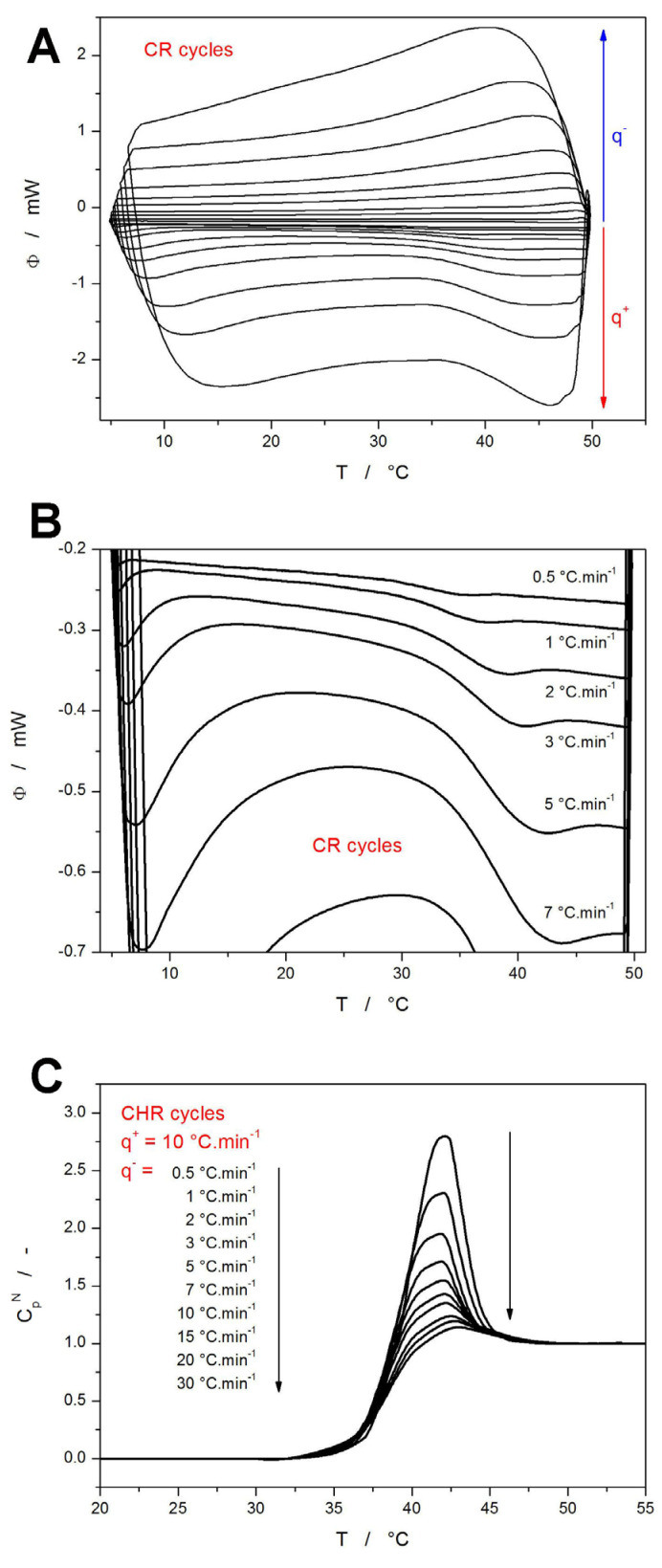
(**A**) A set of CR cycles obtained for the re-melted NIF powder. The exothermic effects evolve in the upward direction. The arrows and symbols *q^−^* and *q^+^* denote the parts of the DSC data in which the cooling and heating steps of the CR cycles are shown, respectively. Absolute magnitudes of *q^−^* and *q^+^* being applied in the corresponding steps of the cyclic program increase in the directions of the given arrows. (**B**) DSC data for the CR cycles zoomed in on the heating steps performed at low *q^+^*. (**C**) A set of CHR cycles obtained for the re-melted NIF powder; only the data corresponding to the already normalized heating curves are shown. The arrows denote the increase in |*q^−^*| in the cooling step preceding to the depicted heating step.

In addition to the simple crystallization measurements (Figure 1 and Figure 2), the dedicated CR and CHR cyclic temperature programs were utilized to study the structural relaxation of NIF. In the first iterations, these were applied to differently sized NIF powders; however, in all cases, the multiple repeated heating and cooling in the glass transition range led to the crystalline phase being formed during the CR and CHR experiments. Note that the full set of cycles needs to be always performed for the same (identical) sample to achieve the required reproducibility of the temperature dependences of heat capacity in the glassy (*c_pg_*-*T*) and undercooled liquid (*c_pl_*-*T*) states. To solve this issue, the samples for the relaxation measurements were prepared directly in DSC by melting and rapidly cooling the NIF powder sealed in the DSC pan. In this way, a uniform, smooth layer of amorphous NIF formed on the bottom of the DSC pan, and the absence of mechanical defects and cracks prevented any crystals from forming. The measured DSC data corresponding to the CR and CHR cycles are shown in Figure 4. A very important conclusion can be reached by comparing the data from Figure 4B (ideal bulk, *q^+^* = 0.5 °C·min^−1^) and Figure 3A (ideal bulk, *q^+^* = 0.5 °C·min^−1^).

Whereas for the equilibrated bulk matter, the glass transition occurs between 29.3 and 38.7 °C (Figure 4B), the as-quenched 500–1000 μm powder exhibits the glass transition between 35.5 and 43.6 °C. This not only unambiguously rules out the influence of thermal gradients as an explanation for the *T*-shifted dependences in Figure 3B, but it also confirms the quenched-in state of the amorphous matter as the reason for the increased *T_g_*. As the tapping/grinding releases the internal stress of the glassy grains, their *T_g_* decreases, which is exactly what was observed experimentally and is quantified in Figure 3B. In contrast to the CR cycles (which are measured mainly to monitor the *T*-shift of *T_g_* with *q*^+^), the data from the CHR cycles need to be examined much more closely, with the main focus being on the height of the relaxation peaks caused by the increasing compactness of the amorphous structure with the ongoing relaxation process. For this reason, the CHR data were normalized according to Equation (2) (see Figure 4C):

(2) $$C_{p}^{N}\left(T\right)=\frac{dT_{f}}{dT}=\frac{C_{p}\left(T\right)-C_{pg}\left(T\right)}{C_{pl}\left(T\right)-C_{pg}\left(T\right)}$$

where *C_p_^N^*(*T*) is the normalized relaxation signal, *C_p_*(*T*) is the measured signal, and *C_pg_*(*T*) and *C_pl_*(*T*) are the extrapolated DSC signals in the glassy and undercooled liquid regions, respectively. Note that for the actual mathematical transformation, the *C_pg_*(*T*) and *C_pl_*(*T*) dependences can be (and were) substituted by the DSC-measured temperature dependences of heat flow obtained for the given temperature regions. The calculations (based on the data from Figure 4) related to the structural relaxation kinetics will be again discussed in Section 4.

### 2.4. Raman and Optical Microscopy Measurements

**Figure 5:**
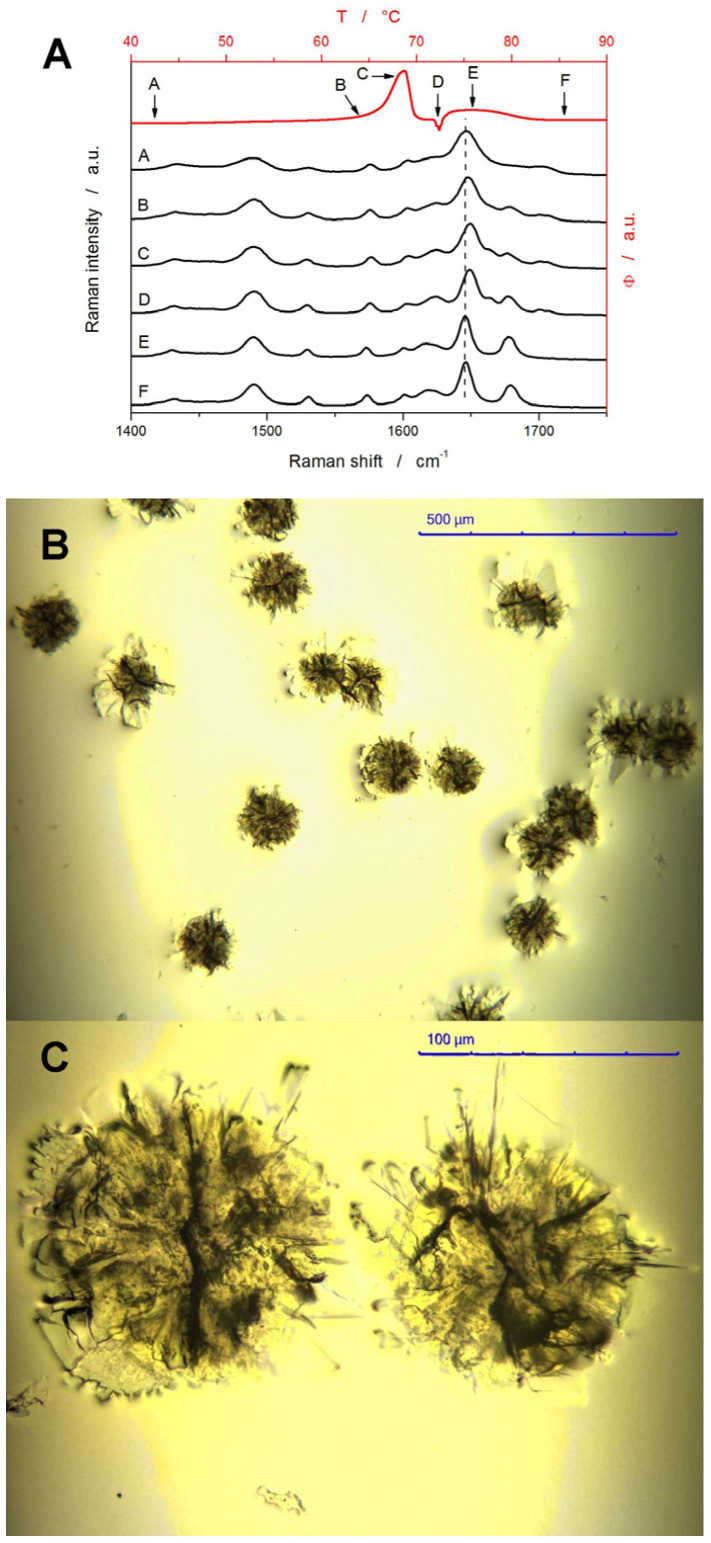
(**A**) DSC curve measured for the amorphous 180–250 μm NIF powder at 1 °C·min^−1^ (red data; top and right axes), where the letters A–F denote specific positions (defined by the corresponding temperatures) on the DSC curve. The Raman spectra then correspond to these characteristic events (as denoted by the arrows and letters) on the DSC curve (black data; bottom and left axes). (**B**,**C**) Optical micrographs of NIF crystals grown at high *q*^+^.

To resolve the polymorphic transitions in the DSC-crystallized NIF samples, Raman microscopy was employed. Figure 5A shows an example measured for the 180–250 μm powder heated at 1 °C·min^−1^. Here, a series of repeated DSC measurements was performed, with each measurement being stopped at one of the specific points/temperatures denoted by letters A–F in Figure 5A; the corresponding spectra are shown below the red DSC curve. For the α_p_ NIF polymorph, the assignment of the main Raman bands is as follows [53]: 810 and 836 cm^−1^~out of plane C-H ring vibration, 967 cm^−1^~dihydropyridine ring (possibly), 1048 cm^−1^~1,2-substituted ring, 1224 cm^−1^~C-C-O ester bond vibration, 1348 cm^−1^~symmetric stretching vibration of NO_2_, 1492 cm^−1^~C=C bond in aromatic circle, 1532 cm^−1^~asymmetric stretching vibration of NO_2_, 1575 cm^−1^~out of plane N-H scission, 1602 cm^−1^~C=C bond in aromatic circle, 1646 cm^−1^~stretching C=C vibration, and 1679 cm^−1^~C=O stretching vibration. The β_p_ polymorph then does not exhibit the 1679 cm^−1^ band but shows two new weak bands at 1664 and 1703 cm^−1^; the position of the main band at 1646 cm^−1^ is also shifted to ~1650 cm^−1^ [28,29,30,31,34]. Analysis of the Raman spectra obtained for the particular stages of the crystallization process shows that initially (stage B), mainly the α_p_ polymorph forms, being quickly (stage C) accompanied by the formation of the β_p_ polymorph. Although identified calorimetrically, no major change in the Raman spectra was observed for the β_p_ → β′_p_ transition (stage D). Consequently, the β’_p_ polymorph spontaneously recrystallizes into the α_p_ polymorph (stage E), which remains the only spectroscopically identifiable form at high temperatures. Very similar courses of the structural/polymorphic development throughout the crystallization experiments were also observed for the other investigated combinations of *q*^+^ and *d_aver_*—the only minor variable was the β_p_ and β′_p_ contents in the initially crystallizing and final materials, respectively. It is worth noting that the combination of DSC and Raman microscopy is very convenient for the study of the crystallization of amorphous drugs, as both techniques provide complementary information about the amorphous and polymorphic crystalline phases. Second large advantage of these techniques is that both require very small amount of sample (contrary to, e.g., X-ray diffraction analysis, XRD), which allows for the investigation of samples with large variety of precisely defined thermal histories, and/or intermediate stages of chemical reactions and physico-chemical transformations. Such a performance would probably be matched only by ex situ microXRD (which is usually significantly less sensitive) or by the in situ synchrotron-sourced macroXRD (which is not commonly accessible).

In addition to Raman microscopy, optical microscopy was used to report on the morphology of the formed crystallites. Since the crystallized powders dominantly exhibit the fine-grained surface crystalline layer (see the Supplementary Materials for micrographs of all as-prepared and DSC-crystallized powders), an amorphous NIF thin film had to be prepared to show the morphology of the fully developed crystallites. The technique for the preparation of the amorphous NIF samples suitable for the optical microscopy measurements was taken from [37], i.e., the NIF powder was melted in between two microscopic slides and then cooled by contact with an aluminum block. The upper slide was gently detached, which resulted in a continuous layer of NIF film being left on the bottom slide. The NIF layer was, as a result of detaching the upper layer, covered with a network of fine cracks. These cracks disappeared (due to the self-healing via viscous flow) at *T_g_*. However, they certainly served as nucleation/growth centers because even despite the very high heating rate that was consequently applied, a number of crystals were still formed on the NIF film (this would not have happened if the film was compact and defects-free). This finding gives insight into the role of the mechanical defects present in the NIF powders, which, too, self-heal above *T_g_*, but their former presence has already stimulated the initiation of crystal growth. Note that the dominant role of mechanical defects is for the low-molecular organic glasses well documented in the literature—see e.g., [34,37]. Technically, impurities might also be responsible for a certain portion of the nucleation/growth behavior, but since the above-described temperature-induced crystal formation is not observed for the smooth NIF surfaces (tested with a droplet solidified on a microscope slide, which does not crystallize at all), the role of impurities and other akin aspects seems to be minor, and the presence of the mechanical defects is crucial for the initiation of the crystal growth.

## 3. Discussion

The present section will be structured as follows: First, the kinetics of structural relaxation will be determined from the CR and CHR cyclic DSC experiments. Second, the advanced methods of kinetic analysis will be used to describe the kinetics of the macroscopic crystal growth, as measured during the simple heating scans. Third, the correlations between the relaxation and crystal growth kinetics will be discussed.

### 3.1. Structural Relaxation Kinetics

Nowadays, the structural relaxation data measured in the glass transition range are standardly described in terms of the Tool–Narayanaswamy–Moynihan (TNM) relaxation model [54,55,56]:

(3) $$\Phi \left(t\right)=exp\left[-{\left(\int_{0}^{t}\frac{dt}{\tau (T,T_{f})}\right)}^{\beta }\right]$$

(4) $$\tau \left(T,T_{f}\right)=A_{TNM}\cdot exp\left[x\frac{∆h^{*}}{RT}+(1-x)\frac{∆h^{*}}{RT_{f}}\right]$$

where the fundamental quantity is the fictive temperature (*T_f_*), which is defined as the temperature of the undercooled liquid with the same structure as that of the relaxing glass at the given time. The fictive temperature is then calculated on the basis of the following quantities: time *t*, temperature *T*, relaxation time *τ*, the apparent activation energy of structural relaxation Δh^*^, the pre-exponential factor *A_TNM_*, the non-linearity parameter *x* (0 < *x* ≤ 1), and the non-exponentiality parameter *β* (0 < *β* ≤ 1). Note that the *C_p_^N^*(*T*) quantity calculated by the transformation of the DSC data is related to Equations (3) and (4):

(5) $$\Phi \left(t\right)=\frac{T_{f}\left(t\right)-T_{f}(\infty )}{T_{f}\left(0\right)-T_{f}(\infty )}$$

where the fictive temperature is an integrated form of the normalized DSC signal (according Equation (2)).

**Figure 6:**
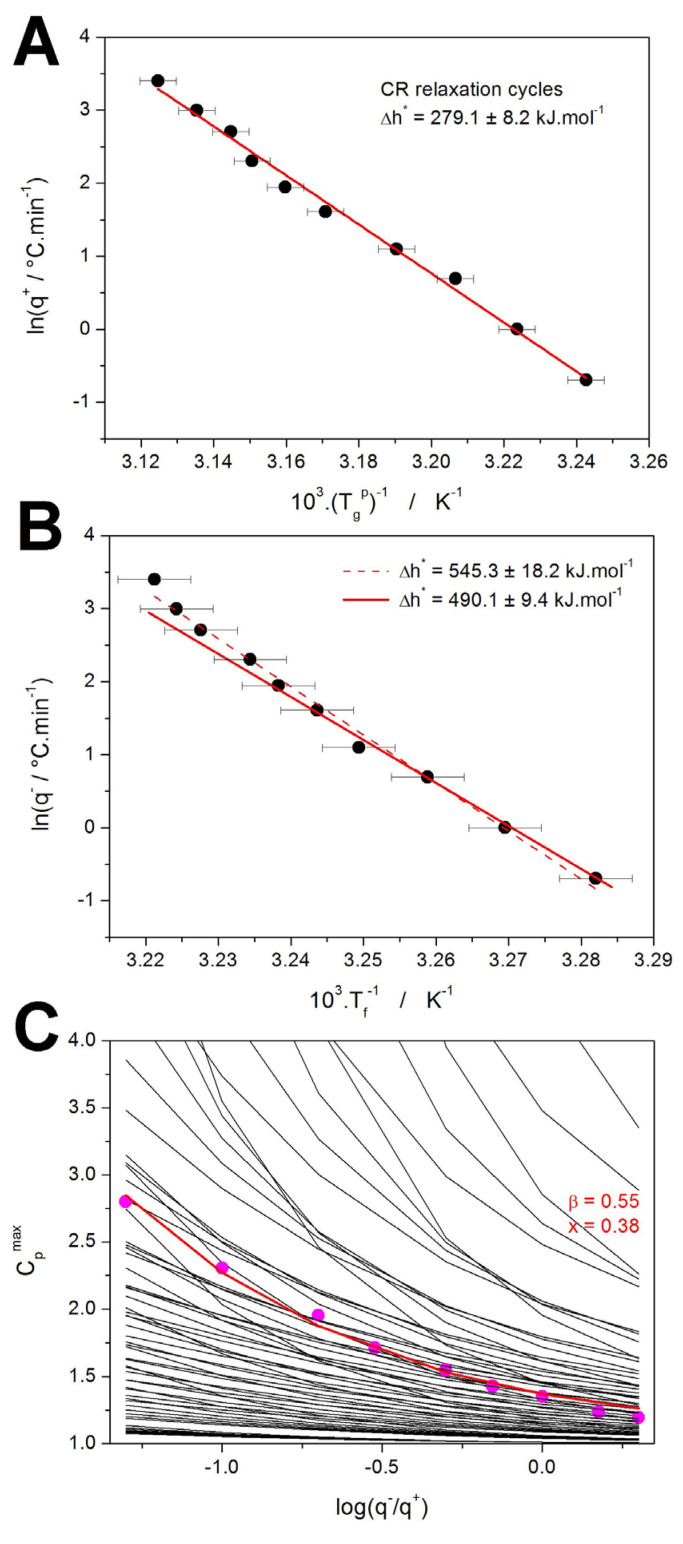
(**A**) Evaluation of Δh^*^ from the CR cycles – points represent experimental data and line is their linear fit. (**B**) Evaluation of Δh^*^ from the CHR cycles – points represent experimental data and lines are their linear fits in two different temperature regions (see text for details). (**C**) The application of the simulation-comparative method to the CHR relaxation measurements of the amorphous NIF. Points correspond to the experimental data. Black solid lines refer to simulated data for the various combinations of the TNM parameters *β* and *x* (both parameters changing with the 0.1 step). Colored line refers to the simulated *β* + *x* combination best fitting the experimental data.

The first quantity to determine is usually the activation energy of structural relaxation Δh^*^. A standard method for this task is based on the evaluation from the CR cycles, which can be expressed by Equaton (6) [50,57]:

(6) $$-\frac{∆h^{*}}{R}={\left[\frac{dln\left|q^{-}\right|}{d(1/T_{p})}\right]}_{q^{-}/q^{+}=const.}$$

where *q^−^* is the cooling rate used in the step preceding the heating scan from which the *T_g_^p^* is determined. This type of evaluation is shown in Figure 6A, where the very good linearity of the depicted dependence indicates an absence of any potential thermal gradients within the system. An alternative method for the determination of Δh^*^ was introduced by Moynihan [58], which is based on the evaluation of *T_f_* from the CHR cycles by the “equal areas method” (explained in Supplementary Materials):

(7) $$-\frac{∆h^{*}}{R}=\frac{dln\left|q^{-}\right|}{d(1/T_{f})}$$

where *q^−^* is again the cooling rate used in the step preceding the heating scan from which the *T_f_* is determined. The evaluation by this method is shown in Figure 6B.

Notably, the Δh^*^ provided by this method is significantly larger (almost double) compared to that provided by Equation (6). This is a common issue observed numerous times for a large variety of structurally and typologically different materials [57]. Whereas, in theory, both methods function equally accurately, in practice, the Moynihan method can be burdened by significant errors as a result of potential inaccuracies in properly normalizing the DSC heat flow data into *C_p_^N^* (due to the unknown *c_pg_*-*T* and *c_pl_*-*T* dependences), which can consequently largely influence the determined *T_f_*. Certain levels of uncertainty can also be assessed from the slight deviation of linear behavior of the data depicted in Figure 6B, which (depending on what part of the dependence is considered to be correct) can result in errors of ~10%. However, the errors in the *T_f_* determination would have to be much larger than that for the present NIF CHR cycles data if equality of the two Δh^*^ values determined from the CR and CHR cycles would be assumed, which we do not find plausible. Hence, an alternative cause for the underlying origin of the inconsistency will be suggested in Section 3.3.

With the knowledge of Δh^*^, a non-linear optimization based on the Leveberg–Marquardt algorithm can be applied to estimate the value of the pre-exponential factor, which was for the present data determined to be ln(*A_TNM_*/s) = −104.1. As is often the case for the low-molecular glasses [46,51,52], the quality of the CHR cycles data is not sufficient [57] to employ the curve-fitting method for the determination of the shape-defining TNM parameters *x* and *β*. In such cases, a very robust and instrumental artifacts-resistant approach, the simulation-comparative method [59], can be used to quite accurately estimate these parameters. This methodology is very robust in comparison to the classic curve-fitting of the whole DSC curves, because it is not influenced by various instrumental artifacts (often manifesting as broadening or skewing of the relaxation peak) that are often present in the DSC data. The simulation-comparative method utilizes the comparison of the experimentally measured and theoretically simulated dependences of the height of the normalized relaxation peak *C_p_^max^* during the CHR cyclic experiments. This is demonstrated in Figure 6C, where the black lines indicate the *C_p_^max^*-log(*q^−^*/*q^+^*) dependences simulated for various combinations of *x* and *β* (further utilizing the Δh^*^ determined from CR cycles, ln(*A_TNM_*/s) = −104.1, and exact temperature program used in the experiments), points show the experimental data and the red line corresponds to the best simulated combination of *x* and *β*. The lines do not represent non-linear fits/dependences, but only linear connections between the individual simulated points. Note that only the theoretical dependences corresponding to the combinations of *x* and *β* changing with the step of 0.1 in the (0.2–1.0) range are shown in Figure 6C. In reality, the simulations were performed with the resolution of 0.01 for each TNM parameter; the best match between the experimentally measured and theoretically simulated series of *C_p_^max^*-log(*q^−^*/*q^+^*) dependences was selected using the least squares method. The obtained values of *x* = 0.38 and *β* = 0.55 indicate a fairly standard structural relaxation behavior of a relatively fragile glass [60]. The following *x* and *β* values were obtained for the other recently studied low-molecular glasses: indomethacin (*x* = 0.32 and *β* = 0.53) [46], nimesulide (*x* = 0.40 and *β* = 0.54) [52], and griseofulvin (*x* = 0.29 and *β* = 0.41) [51]. The NIF glass evidently belongs among the more uniformly relaxing materials with a lower variety of relaxation motions, structural heterogeneity, and cooperativity between the relaxing domains [46,60].

### 3.2. Cold Crystallization Kinetics

The DSC crystallization data obtained for the NIF powders (Figure 1) were first processed with respect to their thermo-kinetic background, and the pure crystal growth signal was obtained by interpolating and subtracting the tangential area-proportional baseline [61]:

(8) $$B\left(T\right)=\left(1-\alpha \left(T\right)\right)\cdot \left(z_{0,r}+z_{1,r}\cdot T\right)+\alpha \left(T\right)\cdot \left(z_{0,p}+z_{1,p}\cdot (T_{f}-T)\right)$$

where *B*(*T*) is the temperature dependence of the baseline curve, *α* is the degree of conversion, *z*_0*,r*_, and *z*_1*,r*_ are the coefficients characterizing the tangent going through the starting point (in the reactants area), *z*_0*,p*_ and *z*_1*,p*_ are the coefficients characterizing the tangent going through the endpoint (in the products area), and *T_f_* is the endpoint temperature. The complex crystallization signal was then described in terms of the standard solid-state kinetic equation [62]:

(9) $$\Phi =∆H\cdot A\cdot e^{-E/RT}\cdot f(\alpha )$$

where *Φ* is the DSC heat flow signal, Δ*H_c_* is the crystallization enthalpy, *A* is the pre-exponential constant, *E* is the activation energy of crystallization, *R* is the universal gas constant, and *f*(*α*) is a function defining the shape of the crystallization peak as a dependence of the degree of conversion *α.* In particular, for the crystal growth from an amorphous state, the two most common *f*(*α*) functions are the nucleation-growth Johnson–Mehl–Avrami [63,64,65,66] (JMA) model (Equation (10)) and the semi-empirical flexible autocatalytic Šesták-Berggren [62] (AC) model (Equation (11)):

(10) $${f(\alpha_{c})}_{JMA}=m\left(1-\alpha_{c}\right){\left[-ln\left(1-\alpha_{c}\right)\right]}^{1-\left(1/m\right)}$$

(11) $${f(\alpha_{c})}_{AC}=\alpha_{c}^{M}{\left(1-\alpha_{c}\right)}^{N}$$

where *m*, *M*, and *N* are the exponents of the two respective kinetic models. The exponent *m* indicates the nucleation conditions and dimensionality of the formed crystallites. The exponents *M* and *N* were suggested as empirical parameters without physical meaning but they can be understood as a reaction order (*N*) and the degree of autocatalysis (*M*), where *M* = 0 and *N* = 1 corresponds to the first order reaction.

**Figure 7:**
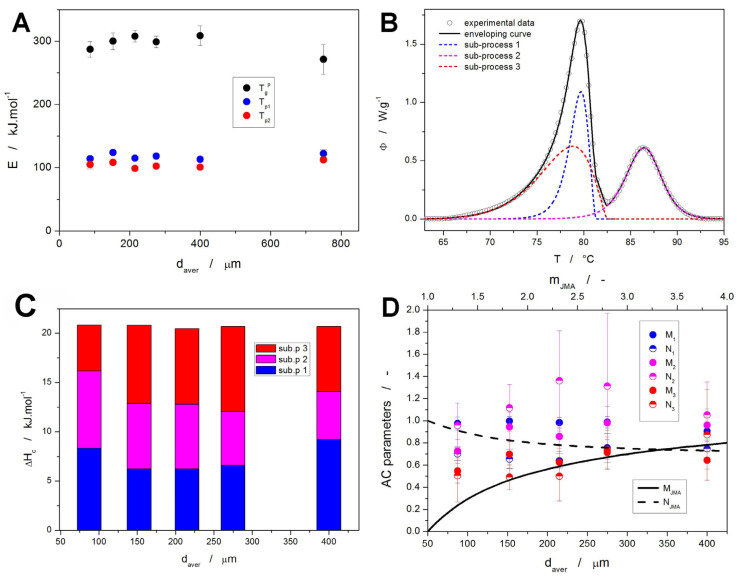
(**A**) Activation energies calculated for the three characteristic temperatures based on the Kissinger plots (Figure 3B–D). (**B**) An example of the NIF crystallization DSC curve (125–180 μm at 10 °C·min^−1^) deconvoluted by means of the sc-MKA method. (**C**) Crystallization enthalpies obtained via sc-MKA method for the NIF powders (the data are averaged over all *q^+^*). (**D**) AC kinetic parameters obtained via sc-MKA method for the DSC crystallization data of the NIF powders (the data are averaged over all *q^+^*). The lines (scaled according to the top axis) indicate the fingerprint *M* + *N* combinations attributed to the various values of the kinetic exponent of the JMA model.

The enumeration of the above-mentioned set of kinetic equations (either Equation (9) + Equation (10) or Equation (9) + Equation (11)) starts (similarly as in the case of structural relaxation) by determining the activation energy of the crystallization process. Here, the Kissinger method [67] (Equation (1)) can be used with great advantage. By evaluating the Kissinger plots (Figure 3B–D) in terms of Equation (1), the particle size-dependent *E* values depicted in Figure 7A were obtained. As is apparent, the crystallization activation energy does not show any prominent trend with *d_aver_*, the *E* values ranging between ~113–125 kJ·mol^−1^ for the first crystallization peak and ~99–112 kJ·mol^−1^ for the second crystallization peak. Interestingly, the inaccurate methodology of applying the Kissinger equation to the structural relaxation data (as described in [68]) provides values of ~270–310 kJ·mol^−1^, which is in agreement with the result of the correct procedure based on the CR cycles (279 kJ·mol^−1^, see Figure 6A). This equality indicates that the relaxation behavior of NIF incidentally hits the sweet spot of the Δh^*^ + *x* + *β* combination [68], for which the error is negligible.

With the knowledge of the *E* values, the recently developed single-curve multivariate kinetic approach [69] (sc-MKA, a non-linear optimization method based on the Levenberg–Marquardt algorithm) was used:

(12) $$RSS=\sum_{j=1}^{n}\sum_{{First}_{j}}^{{Last}_{j}}w_{j,k}{\left({Yexp}_{j,k}-{Ycal}_{j,k}\right)}^{2}$$

(13) $$w_{j}=\frac{1}{{\left|{\left[d\alpha /dt\right]}_{max}\right|}_{j}+{\left|{\left[d\alpha /dt\right]}_{min}\right|}_{j}}$$

where *RSS* is the sum of squared residue, *n* is a number of measurements, *j* is an index of the given measurement, *First_j_* is the index of the first point of the given curve, *Last_j_* is the index of the last point of the given curve, *Yexp_j,k_* is the experimental value of the point *k* of curve *j*, *Ycal_j,k_* is the calculated value of the point *k* of curve *j* (calculated in accordance with Equation (9)), and *w_j_* is a weighting factor for curve *j*. Note that the sc-MKA method utilizes fixed *E* values to decrease the number of mutually dependent variables in Equation (9), which allows curve-fitting to be applied to each data curve separately and determine the *q^+^*-based trends in the data. In the present study, the *E* values obtained by the Kissinger method (Figure 7A) were used. Since the DSC crystallization signal is complex, consisting of several overlapping peaks, the following set of equations replaces Equation (9) in Equation (12):

(14) $$\frac{d\alpha_{c,x}}{dt}=A_{x}\cdot exp\left(-\frac{E_{x}}{RT}\right)\cdot \alpha_{c,x}^{M}\cdot {\left(1-\alpha_{c,x}\right)}^{N}$$

(15) $$1=\sum_{x=1}^{n}\alpha_{c,x}$$

where “*x*” is the index of the given individual sub-process, and “*n*” is the number of sub-processes (three in the present case). Regarding the transformation mechanism, a combination of three independent processes was found to be the minimum needed for the accurate description of the crystallization data. Note that the complex crystallization from an amorphous matrix very often proceeds as a sum of independent processes due to the growth proceeding from individually formed nuclei. The kinetic analyses of the data obtained for the 500–1000 μm powder were not performed due to the irregularities of the crystallization peaks, where each of the few measured grains crystallized at lower *q^+^* individually (with the preferential crystallization centers playing an overwhelming role).

A typical example of the NIF crystallization signal deconvoluted using the set of Equations (11)–(15) is shown in Figure 7B. Regarding the asymmetry of the crystallization peaks, extensive testing has shown that the absolute majority of the data cannot be accurately described by the physically meaningful JMA model and that the more flexible AC model will have to be used instead (as depicted in Figure 7B). The sets of kinetic parameters resulting from the sc-MKA optimization are included in the Supplementary Materials; their values averaged over all applied *q^+^* are shown in Table 1 and depicted in Figure 7C,D. Based on the combination of the information from the temperature-resolved Raman microscopy (Figure 5A) and deconvoluted crystallization data (Figure 7B), the sub-process no. 1 should correspond to the formation of the α_p_ polymorph, the sub-process no. 3 should represent crystallization of the β_p_ polymorph, and sub-process no. 2 should indicate the recrystallization from the β_p_ phase into the α_p_ phase. Considering the distribution of the crystallization enthalpies associated with the individual crystal growth sub-processes (shown in Figure 7C), it is clear that for the fine powders, practically all formed β_p_ phase transforms into the α_p_ phase after/during the initiation of the β_p_ → β_p_′ transition. As the powder coarseness increases, a certain portion of the β_p_/β_p_’ phase remains untransformed into the α_p_—presumably inside the NIF grains, where the surface mobility facilitating the β_p_/β_p_′ → α_p_ transformation has limited reach. Note that using the Raman microscopy, no β_p_/β_p_′ phase was detected on the surface of the fully crystallized coarse NIF grains. The amount of the untransformed β_p_/β_p_′ phase in the coarse NIF powders also largely increased with decreasing *q^+^*. The average values of the AC kinetic exponents are for the three sub-processes shown in Figure 7D, where the lines indicate the *M* and *N* values characteristic of the JMA processes (no correspondence between the present data and the JMA kinetics was found). Nonetheless, it is apparent that the crystal growth sub-processes manifesting within the first crystallization peak exhibit a clearly negative asymmetry, whereas the sub-process no. 2 (the β_p_/β_p_′ → α_p_ recrystallization) has significantly more positive asymmetry (skewing to lower *T* represented by higher *N* and *M* values), which indicates the higher degree of autocatalysis associated with the already existing β_p_/β_p_′/α_p_ interfaces. Note that the autocatalytic behavior is characterized by higher *M* values (the transformation rate d*α*·d*t*^−1^ increases as the amount of products represented by *α* increases), and the higher *N* values indicate typically overall faster transformations (the positive asymmetry typical for the higher order reactions indicates a fast transformation onset with prolonged process termination).

### 3.3. Correlation Between the Relaxation and Crystallization Kinetics

**Figure 8:**
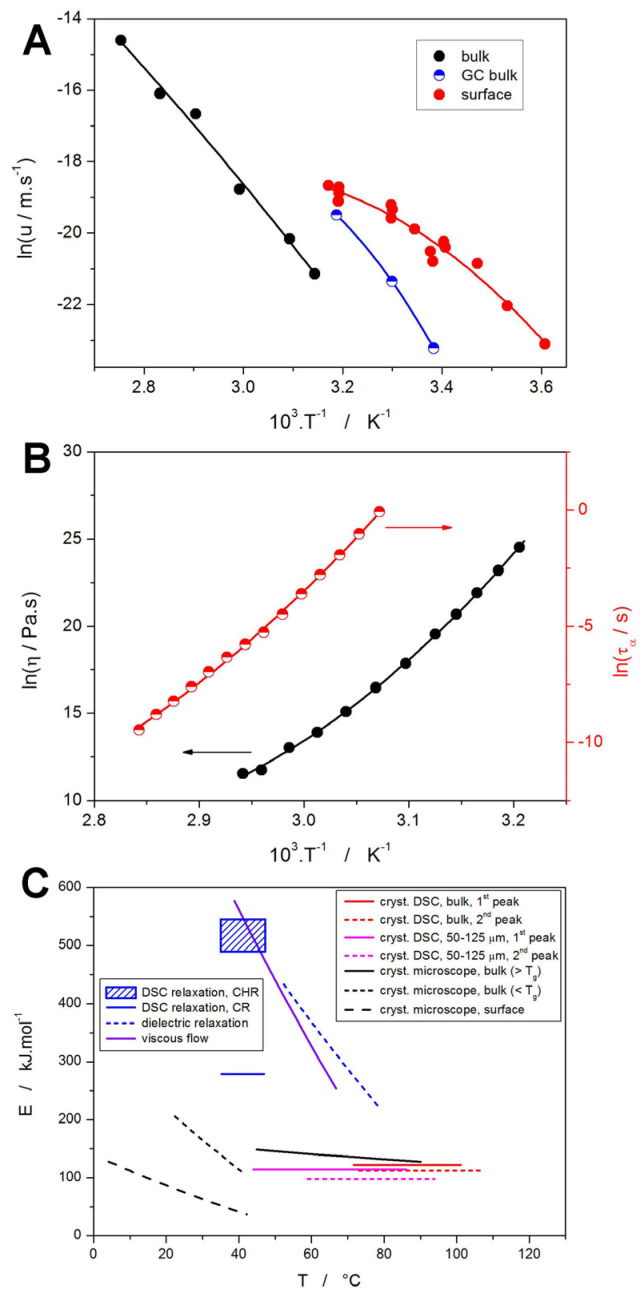
(**A**) Temperature dependences of different crystal growth modes (taken from [70]). Points represent experimental data, lines indicate second-order polynomial fits. (**B**) Temperature dependences of dynamic viscosity (black data and left Y axis) and dielectric relaxation time (red data and right Y axis) taken from [42,71]. Points represent experimental data, lines indicate second-order polynomial fits. (**C**) Comparison of temperature dependences of activation energies determined for different processes occurring in amorphous NIF.

As was shown in the cases of several other recently studied low-molecular glasses [46,51,52], interesting correlations can be found between certain thermo-kinetic processes based on the equality of their activation energies. In order to map the largest possible variety of the thermally induced processes associated with amorphous NIF, several series of literature data were taken, and the activation energies of the monitored processes were calculated. The microscopically observed crystal growth rates were taken from [70]; in particular, the data for the above-*T_g_* growth in bulk, the below-*T_g_* growth in bulk (the rapid glass-crystal GC growth), and the below-*T_g_* growth on the surface analyzed, as shown in Figure 8A (*T_g_* of NIF is at ~40 °C, i.e., X coordinate = 3.193). The growth rate data were fit by the second-order polynomials (solid curves in Figure 8A). The activation energy of the microscopic crystal growth was then calculated from the derivation of the polynomial dependences according to the following:

(16) $$\frac{dln\left(u_{G}\right)}{d\left(1/T\right)}=-\frac{E_{u}}{R}$$

where *u* is the crystal growth rate, and *E_u_* is the corresponding activation energy. Similar analyses were also performed for the complex dynamic viscosity *η* from [42] and for the dielectrically measured above-*T_g_* relaxation time *τ_α_* from [71]; the data are depicted in Figure 8B together with their fits by the second-order polynomial functions. The corresponding equations for the evaluation of the activation energies *E_η_* and *E_τα_* are as follows:

(17) $$\frac{dln\left(\eta \right)}{d\left(1/T\right)}=\frac{E_{\eta }}{R}$$

(18) $$\frac{dln\left(\tau \right)}{d\left(1/T\right)}=\frac{E_{\tau }}{R}$$

The comparison of the temperature dependences of the activation energies is shown in Figure 8C. Starting with the process of structural relaxation, it is often considered to be a continuation of the viscous flow, with the two activation energies being similar [72,73]. Such similarity is particularly apparent for the activation energies obtained for the viscous flow, dielectric relaxation (calculated from *τ*_α_, where “α” is commonly used as a denotation of the main relaxation process involving the reorganization of whole relaxation domains and/or relaxing entities, i.e., molecules and molecular clusters in the case of NIF). Considering the present DSC relaxation data, interestingly, the Δh^*^ values obtained from the CHR cycles (which are usually thought to be less accurate and potentially burdened with larger evaluation errors) are in good agreement with *E_η_* as well. On the other hand, Δh^*^ values certainly accurately determined from the CR cycles are evidently in disagreement with the activation energy of viscous flow, being significantly closer to the *E* values for the crystal growth. As was already mentioned in Section 3.1, such a large discrepancy has to have an underlying physico-chemical meaning. Since a relatively high (and constant) *q^+^* = 10 °C·min^−1^ is used for CHR cycles, it can be assumed that a majority of the relaxation motions released during the narrow temperature window near the corresponding *T_g_* (note that a large range of *T_f_* values was still achieved during the different cooling rates. Hence, the full variety of relaxation scales and motions should be present during the glass transition). As such, practically, the whole manifestation of the structural relaxation process should be captured in the determined *T_f_* value, which is conformable to the viscous flow manifestation. In contrast, the large variety of *q^+^* being applied during the CR cycles may lead to an anomalous (with respect to the standard TNM philosophy) gradual antecedent release of the frozen-in glassy structure, spreading the manifestation of the relaxation motions over a broader temperature range (during the whole *q^+^* series). This would, naturally, lead to a significantly lower value of Δh^*^, as observed experimentally. An alternative explanation may be associated with the occurrence of the so-called “upper relaxation peaks” [74,75], which would, during the CR cycles, capture only a certain portion of the relaxation motions, corresponding to the relaxation time scale present for the given temperature range. Either way, this issue deserves attention in further research. Note that the upper relaxation peaks represent a typical structural relaxation feature (associated with the main, i.e., α relaxation, and reproduced by the TNM model), which shows how the non-linearity concept is fully integrated into the relaxation motions only at deeper relaxation states [74,75].

Moving to the correlation between the microscopically and macroscopically observed crystal growth activation energies, the data for the DSC studied powders, and bulk correspond to the standard microscopic observation of the above-*T_g_* crystal growth and its activation energy. The significantly lower *E* associated with the enhanced surface growth is not consistent with what was observed via DSC. On the other hand, the initiation of crystal growth in fine NIF powder has almost exactly the same *E* as *E_u_* determined for the GC growth in the same temperature range. This indicates that, although the crystal growth may be initiated at the surface or via the GC growth along the micro-cracks (a more probable option), the majority of the crystal growth within the amorphous NIF grains proceeds through the standard bulk growth mechanism. It may also be of considerable interest to further explore the possible dominance of the GC growth during the long-term storage at laboratory temperatures (just below *T_g_*), which could be the basis for reliable determination of the macroscopic kinetics of this growth mode (data akin to those shown in Figure 3A).

Lastly, the relationship between the structural relaxation and crystal growth processes themselves will be discussed. For the low-molecular glasses, the Johari–Goldstein (or “β” relaxation) movements are believed to be decisive for the initiation of crystal growth [48,76,77]. Note that despite the different relaxation modes (α, β, or γ relaxations) being primarily observed in the frequency domain (usually via dielectric measurements), these modes represent real relaxation movements, which are present during the standard (non-modulated) non-isothermal and isothermal experiments performed in the respective temperature ranges. As such, the weaker (β, γ, …) relaxation modes are usually not observable via the DSC measurements (such as performed in the present work), but their influence (such as the possible initiation of the nucleation and/or crystal growth) can still manifest and be observable macroscopically. For amorphous NIF, the activation energy of the β-relaxation processes was reported to be *E_τβ_* = 51 kJ·mol^−1^ in the −50–20 °C temperature range [34], which does not match any crystal growth (macro- or microscopic) recorded for NIF. Regarding the main structural relaxation process (the “α” relaxation near and above *T_g_*), the recently introduced Transition Zone Theory (TZT) [45] needs to be reminded, which promotes the idea of the cooperatively rearranging regions to be the key factor for the crystal growth. As suggested in [46], the combination of the TNM parameters *x* and *β* can be used to assess the degree of cooperativity in the amorphous phase, which can be subsequently potentially related to the above-*T_g_* cooperativity needed to initiate the crystal growth. For NIF, these TNM parameters are *x* = 0.38 and *β* = 0.55, which indicates a relatively moderate degree of regional cooperation (the dynamic cooperativity during the relaxation motions increases with increasing *β* and decreasing *x*). A significantly higher cooperativity (understood as the interconnection of the rearranging regions during the structural relaxation) is expected for indomethacin (*x* = 0.32 and *β* = 0.53) [46] and griseofulvin (*x* = 0.29 and *β* = 0.41) [51]. However, both these materials exhibit lower bulk crystal growth rate at *T_g_* (approx. ln(u/m·s^−1^) = −24 [46,51]) compared to NIF (approx. ln(u/m·s^−1^) = −22, see Figure 8A). This would suggest (if the correlation between the structural relaxation and crystal growth is relevant) that a certain limit in the complexity and magnitude of the cooperating regions may exist, which slows down the crystal growth. Such an assumption may be logical from the following point of view: the larger the necessary cooperating unit/region, the more difficult and energetically more demanding achieving the required configuration is.

## 4. Materials and Methods

The amorphous NIF was prepared by the standard melt-quench technique from its as-purchased polymorphic form α_p_ (Sigma-Aldrich, Prague, Czech Republic: 99.5% purity). In particular, the purchased NIF powder was placed in a glass vial and melted using an oil bath annealed to 175 °C (just above the NIF melting point); the glass vial with the melt was consequently quenched in 10 °C water. The NIF preparation, as well as the consequent manipulation, storage, and measurements, were performed in the dark because NIF is highly light-sensitive [78,79]. The overall exposure to light (standard laboratory lighting conditions) did not exceed 10 min. The prepared NIF ingots were powdered by gentle tapping using an agate mortar and pestle. The amorphous powders were sieved, using Retsch sieves (Retsch/Verder, Prague, Czech Republic) with defined mesh size, to prepare the following particle size fractions: 50–125 μm, 125–180 μm, 180–250 μm, 250–300 μm, 300–500 μm, and 500–1000 μm. Each powder fraction was prepared and processed separately because even storage at 0 °C (~40 °C below the glass transition temperature, *T_g_*) for 24 h led to an alteration of the material: the low-temperature nuclei and/or crystallites were formed. In addition, for the fine NIF powders (50–125 μm and 125–180 μm), which are most prone to the rapid spontaneous formation of the crystalline phase even during the low-*T* storage, three repeated preparations of the same powder had to be performed to cover all the measurements in a time-frame with non-detectable changes in the crystallization behavior (this will be discussed at length in Section 3).

The thermo-kinetic characterization of the prepared NIF powders was conducted using a differential scanning calorimeter (DSC) Q2000 (TA Instruments, New Castle, DE, USA) equipped with an autosampler, an RCS90 cooling accessory, and T-zero technology. The DSC was calibrated for temperature and heat flow using the In, Zn, and H_2_O standards. The DSC measurements were performed in the hermetically sealed low-mass Al pans (i.e., with a static air atmosphere); the sample masses varied between 2 and 3 mg (being accurately weighted to 0.01 mg). The structural relaxation measurements were performed by applying two types of temperature programs, and for the constant ratio (CR) and constant heating rate (CHR) cycles, the sample is repeatedly cooled and heated through the glass transition region [57]. The applied cooling rates were *q^−^* = 0.5, 1, 2, 3, 5, 7, 10, 15, 20 and 30 °C·min^−1^. For the CHR cycles, all heating steps were performed at *q^+^* = 10 °C·min^−1^; during the CR cycles, the heating rates were of the same absolute magnitude as the rate of the preceding cooling. Since the finer powders exhibited an increased tendency towards crystallization even at temperatures close to *T_g_*, the measurements were performed for the ideal bulk sample (a melt-quenched droplet formed directly in the DSC pan), which did not exhibit any sign of crystallization in the temperature range of the relaxation measurements (−10–55 °C). A graphical representation of both types of cyclic relaxation temperature programs is shown in the Supplementary Materials. The crystallization of the amorphous NIF powders was performed as a series of simple heating scans from 0 to 200 °C; the applied heating rates were *q^+^* = 0.5, 1, 2, 5, 10, and 20 °C·min^−1^.

The amorphous character of the quenched NIF ingot and the consequently prepared powders was confirmed using the DXR2 Raman microscope (Nicolet/Thermo Fisher Scientific, Prague, Czech Republic), equipped with a 785 nm excitation diode laser (30 mW, laser spot size of 3.1 μm) and CCD detector. The experimental conditions for the Raman measurements were: a 10 mW laser power on the sample, 5 s duration of a single scan, and 50 scans summed in one spectrum. The same set of conditions was also used to investigate the NIF crystalline phases formed during the crystallization experiments. The NIF crystallites formed non-isothermally as well as during the long-term low-*T* annealing near *T_g_* were also investigated microscopically, using the optical microscope iScope PLMi (Euromex, Arnhem, The Netherlands), equipped with 40× and 80× high-quality objectives and a Moticam visual camera (Motic, Xiamen, China).

## 5. Conclusions

The thermally induced processes in amorphous NIF, namely the structural relaxation and crystal growth, were studied using DSC, optical, and Raman microscopies. The structural relaxation (i.e., the glass transition) kinetics was described in terms of the TNM concept, modeling the relaxation response with the following set of the TNM parameters: ∆h^*^ = 279 ± 8 kJ·mol^−1^, ln(*A*/s) = −104.1, *x* = 0.38 and *β* = 0.55. The ∆h^*^ ≈ 500 kJ·mol^−1^ determined by the alternative method was found to be close to the activation energy of viscous flow in the glass transition range. The large discrepancy between the results of the two evaluation methodologies is assumed to have an underlying physico-chemical cause, which is probably associated with only a certain portion of the relaxation motions being captured or manifesting within the main relaxation peak during the CR relaxation experiments.

The complex macroscopic crystallization was found to manifest as an overlap of two exothermic peaks, where (at least under certain conditions) an additional endothermic signal can occur in between them. Based on the combination of Raman spectroscopy and kinetic deconvolution of the DSC signal, the crystal growth is initiated by the formation of the α_p_ polymorph, closely followed by the formation of the β_p_ crystals, which; however, are transformed (the endothermic signal indicates an enantiotropic polymorphism) into the β_p_′ phase. The second exothermic DSC peak then indicates a recrystallization of the β_p_/β_p_′ crystals into the α_p_ polymorph. Kinetics-wise, all three crystallization processes were described in terms of the autocatalytic model. However, the initial formation of the α_p_ and β_p_ phases was much closer to the standard nucleation-growth behavior compared to the clearly highly autocatalyzed β_p_/β_p_′ → α_p_ recrystallization. The activation energy of the macroscopic DSC-monitored crystallization was very close to that of the bulk-located microscopic crystal growth. Nonetheless, the very initiation of the macroscopic crystal growth may be associated with the sub-*T_g_* GC growth—this assumption is based not only on the even better correlation of the activation energies but also on the very low thermal stability of the powdered NIF, which even during short storage ~10–20 °C below *T_g_* exhibits the amorphous-to-crystalline transformation.

Based on the correlations between the structural relaxation and crystal growth processes being sought within the TZT concept, a certain limit in the complexity and magnitude of the cooperating regions may exist, which can slow down the crystal growth.

## Figures and Tables

**Table 1:** Kinetic parameters from Equations (14) and (15) obtained by means of sc-MKA. The indices correspond to the particular sub-processes as identified in Figure 7B. The pre-exponential factors are in “s^−1^”, activation energies are in “kJ·mol^−1^”, enthalpies are in “kJ·mol^−1^”.

Powder	50–125		125–180		180–250		250–300		300–500	
	Mean	St.Dev.	Mean	St.Dev.	Mean	St.Dev.	Mean	St.dev.	Mean	St.Dev.
log(*A*_1_)	16.22	0.20	17.72	0.09	15.96	0.08	16.53	0.15	15.53	0.10
*E*_1_	114.30	0.00	124.00	0.00	114.90	0.00	118.10	0.00	113.30	0.00
*N*_1_	0.70	0.06	0.66	0.09	0.64	0.06	0.75	0.13	0.75	0.08
*M*_1_	0.97	0.06	1.00	0.04	0.98	0.05	0.99	0.05	0.90	0.04
log(*A*_2_)	14.01	0.23	14.96	0.24	12.86	0.17	13.85	0.37	13.45	0.36
*E*_2_	104.90	0.00	108.30	0.00	98.90	0.00	102.50	0.00	100.80	0.00
*N*_2_	0.96	0.20	1.12	0.21	1.36	0.45	1.31	0.66	1.05	0.30
*M*_2_	0.72	0.11	0.94	0.14	0.86	0.11	0.98	0.15	0.96	0.15
log(*A*_3_)	15.72	0.15	17.28	0.16	15.45	0.14	16.13	0.18	15.23	0.19
*E*_3_	114.30	0.00	124.00	0.00	114.90	0.00	118.10	0.00	113.30	0.00
*N*_3_	0.50	0.24	0.49	0.11	0.50	0.22	0.74	0.17	0.87	0.41
*M*_3_	0.55	0.11	0.70	0.12	0.62	0.15	0.71	0.14	0.64	0.08
Δ*H*_1_/Δ*H*	0.40	0.10	0.30	0.04	0.30	0.13	0.32	0.04	0.44	0.15
Δ*H*_2_/Δ*H*	0.38	0.06	0.32	0.03	0.32	0.04	0.26	0.05	0.24	0.10
Δ*H*	20.8	2.6	20.8	2.8	20.5	3.5	20.7	3.3	20.7	3.3
r	0.9996	0.0003	0.9996	0.0003	0.9962	0.0045	0.9973	0.0063	0.9979	0.0042

## Data Availability

The original data presented in the study are available on request from the author.

## Supplementary Materials

The following supporting information can be downloaded at: https://www.mdpi.com/article/10.3390/molecules30010175/s1, Table S1—Base thermal characteristics obtained from the DSC measurements of amorphous NIF powders. Figure S1—Graphical representation of the Moynihan’s equal areas method. Table S2—The results of the sc-MKA method applied to the DSC crystallization data of NIF powders. Figure S2—graphical representation of the CR and CHR cyclic temperature programs. Figure S3—optical micrographs corresponding to the as-prepared and DSC-crystalized NIF powders. Figure S4—alternative depictions of crystallization and melting enthalpies in J·g^−1^ (variants of Figure 3E,F and Figure 7C).
