# Supplementary material for: Thermally Induced Phenomena in Amorphous Nifedipine: The Correlation Between the Structural Relaxation and Crystal Growth Kinetics
Source: Molecules. 2025 Jan 4;30(1):175. doi: 10.3390/molecules30010175 (PMC11720989; doi:10.3390/molecules30010175)
Supplement: Supplementary file 1 [file molecules-30-00175-s001.zip › molecules-3379981-supplementary.pdf]

Supplemental online material

Table S1 – *Base thermal characteristics obtained from the DSC measurements of amorphous NIF powders.*

powder	q^+	T_g	T_g^p	T_{ons}	T_{p1}	T_{p2}	ΔH_c	T_m^{ons}	T_m^p	ΔH_m
50 - 125	0.5	30.142	33	51	53.4	55.5	61.43	164.75	167.43	99.49
	1	33.01	35.13	55.46	58.13	64.5	53.81	163.41	167.81	98.74
	2	36.09	37.66	61.82	64.91	70.19	64.91	164.99	167.99	99.76
	5	36.21	39.55	67.53	70.72	77.45	69.82	165.95	168.4	100.4
	10	37.7	41.29	72.6	76.65	86.93	66.83	165.88	169.29	97.09
	20	41.45	43.34	78.8	83.4	90.87	70.97	166.52	170.34	98.23
powder	q^+	T_g	T_g^p	T_{ons}	T_{p1}	T_{p2}	ΔH_c	T_m^{ons}	T_m^p	ΔH_m
125-180	0.5	30.13	34.2	54.98	57.57	60.15	64.65	166.54	169.1	103.6
	1	33.71	36.39	59.98	62.44	64.82	55.31	166.43	169.41	107.2
	2	35.92	38.45	65.24	67.68	72.24	62.53	166.63	169.55	101.7
	5	37.39	40.66	71.15	74	79.03	68.08	171.22	172.42	112.9
	10	39.18	42.71	75.57	79.72	86.29	68.09	167.84	170.77	105.7
	20	40.97	43.82	81.62	85.89	92.45	76.7	168.48	171.87	110.8
powder	q^+	T_g	T_g^p	T_{ons}	T_{p1}	T_{p2}	ΔH_c	T_m^{ons}	T_m^p	ΔH_m
180-250	0.5	32.76	35.45	59.66	62.14	67.66	51.7	163.5	167.81	99.46
	1	34.5	37.5	65.91	68.66	75.87	65.33	164.23	168.32	100.1
	2	37.28	38.76	70.68	73.6	82.66	65.91	164.85	168.59	98.97
	5	37.9	41.13	77.87	81.12	91.82	69.68	165.48	169.26	103.2
	10	39.53	43.18	83.67	87.61	99.4	71.71	166.68	170.17	105.4
	20	43.55	45.24	87.56	93.77	105.4	72.06	166.74	171.12	93.63
powder	q^+	T_g	T_g^p	T_{ons}	T_{p1}	T_{p2}	ΔH_c	T_m^{ons}	T_m^p	ΔH_m
250-300	0.5	34.5	36.24	60.36	62.14	64.82	50.96	167.64	169.88	106.9
	1	36.08	38.29	65.43	67.39	73.03	56.24	167.92	170.11	108.9
	2	38.72	40.03	70.13	72.38	77.61	67.86	168.01	170.28	111.1
	5	39.25	42.08	75.05	78.7	85.19	67.89	168.21	170.67	108.4
	10	40.54	44.13	81.36	85.81	94.19	67.58	168.79	171.64	102.4
	20	44.64	46.5	87.57	92.9	101.45	75.65	169.43	172.72	110.7
powder	q^+	T_g	T_g^p	T_{ons}	T_{p1}	T_{p2}	ΔH_c	T_m^{ons}	T_m^p	ΔH_m
300-500	0.5	34.82	37.82	61.3	63.66	66.56	49.48	167.4	169.95	105.9
	1	36.71	39.24	66.44	68.79	74.77	53.44	168.06	170.3	103.9
	2	39.75	41.13	71.11	73.96	79.66	64.33	168.5	170.43	108.7
	5	40.24	43.18	75.31	81.23	88.51	66.92	168.66	171.11	105.5
	10	41.61	44.92	83.16	88.3	95.61	73.58	169.19	171.79	111.4
	20	44.61	47.76	89.19	95.86	104.61	73.69	170.81	173.46	108.4

powder	q^+	T_g	T_g^p	T_{ons}	T_{p1}	T_{p2}	ΔH_c	T_m^{ons}	T_m^p	ΔH_m
500-1000	0.5	37.53	40.44	68.36	71.73	73.16	61.79	170.3	171.32	115.9
	1	38.92	41.07	72.55	74.63	77.71	66.86	170.02	171.04	118
	2	40.19	43.47	77.31	80.92	85.66	70.26	170.67	171.75	112
	5	42.21	46.76	85.75	90.39	93.12	74.24	170.88	173.26	110.4
	10	43.1	47.64	90.39	94.76	99.43	74.45	172.17	173.72	112.8
	20	45.72	51.4	94.63	101.1	106.98	77.66	172.37	175.26	115.3

Figure S1 – *Graphical representation of the Moynihan's equal areas method.*

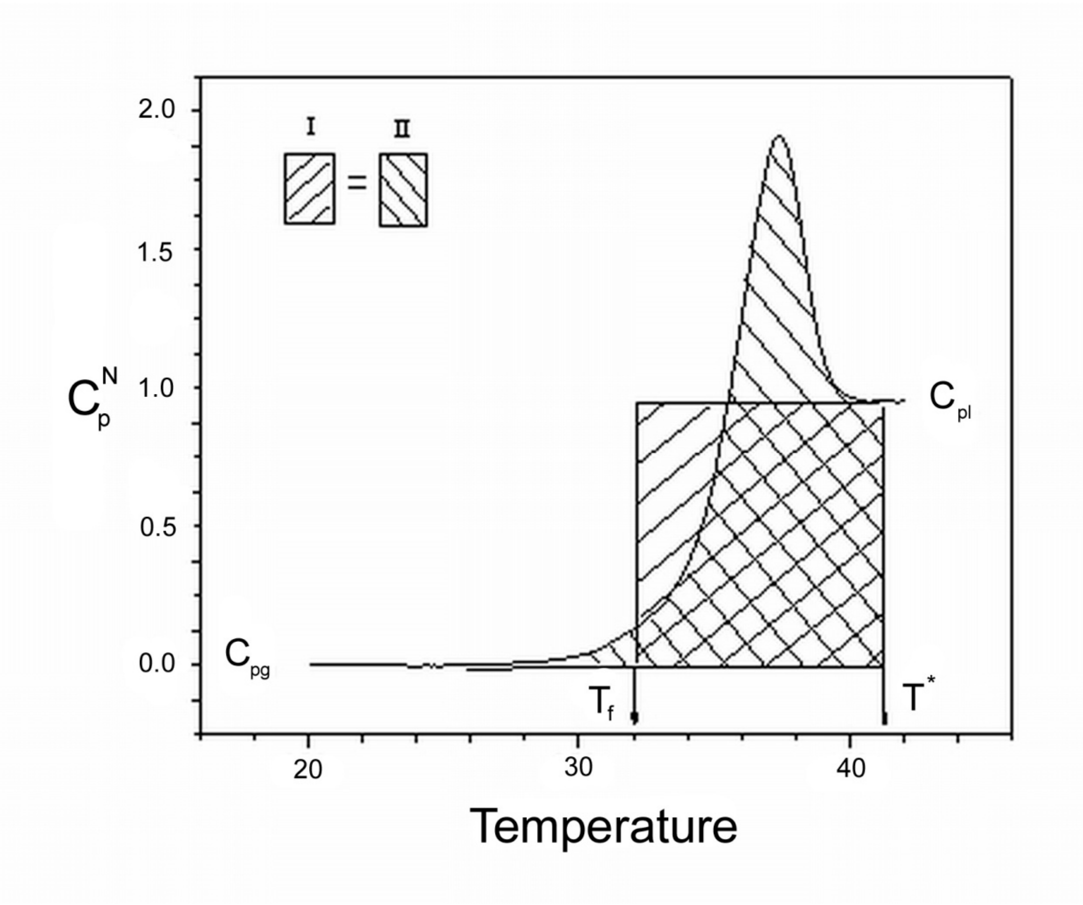

Table S2 – *The results of the sc-MKA method applied to the DSC crystallization data of NIF powders.*

50 – 125 µm						
q^+	0.5	1	2	5	10	20
$\log(A_1)$	16.61108	16.21726	16.07724	16.14603	16.13793	16.1286
E_1	114.3	114.3	114.3	114.3	114.3	114.3
N_1	0.71578	0.71685	0.6064	0.68519	0.68983	0.79353
M_1	1.08685	0.99073	0.95979	0.93186	0.93569	0.94401
$\log(A_2)$	14.43758	13.88043	13.93524	14.07167	13.91808	13.80624
E_2	104.9	104.9	104.9	104.9	104.9	104.9
N_2	0.78186	0.7479	1.13295	1.26169	0.92056	0.89957
M_2	0.83517	0.69899	0.73803	0.79265	0.7523	0.52812
$\log(A_3)$	15.84363	15.55615	15.62165	15.95344	15.71288	15.63753
E_3	114.3	114.3	114.3	114.3	114.3	114.3
N_3	0.22245	0.27432	0.40329	0.80225	0.63848	0.68753
M_3	0.54031	0.35569	0.5151	0.68013	0.60195	0.59076
$\Delta H_1/\Delta H$	0.32998	0.47018	0.49405	0.49332	0.31383	0.29648
$\Delta H_2/\Delta H$	0.48785	0.37548	0.35505	0.34149	0.30222	0.3928
ΔH	60.02713	46.53704	60.25898	63.58853	62.3003	68.49157
r	0.999283	0.999585	0.999242	0.999595	0.999925	0.999919

125 – 180 µm						
q^+	0.5	1	2	5	10	20
$\log(A_1)$	17.85998	17.73347	17.75048	17.72093	17.60956	17.63822
E_1	124	124	124	124	124	124
N_1	0.68043	0.72883	0.56739	0.5676	0.61335	0.77514
M_1	1.07068	0.98209	1.01537	0.98471	0.94121	0.97735
$\log(A_2)$	15.15411	15.02412	15.03406	15.15007	14.86001	14.53043
E_2	108.3	108.3	108.3	108.3	108.3	108.3
N_2	0.96341	0.96219	1.29088	1.45268	1.08735	0.93687
M_2	1.05626	0.9467	1.0064	1.03809	0.9236	0.6879
$\log(A_3)$	17.3237	17.53094	17.35824	17.19903	17.13645	17.10639
E_3	124	124	124	124	124	124
N_3	0.33101	0.47651	0.46852	0.45046	0.56235	0.66068
M_3	0.76354	0.8585	0.79139	0.62079	0.56778	0.57734
$\Delta H_1/\Delta H$	0.31812	0.33289	0.30173	0.33977	0.27667	0.22922
$\Delta H_2/\Delta H$	0.32352	0.34358	0.30394	0.28159	0.29465	0.36073
ΔH	61.5838	45.90207	56.707	63.54977	62.95117	70.21816
r	0.999396	0.999819	0.999248	0.999321	0.999614	0.999941

180 – 250 µm						
q^+	0.5	1	2	5	10	20
$\log(A_1)$	15.9333	15.91214	16.04718	16.04271	15.96967	15.84261
E_1	114.9	114.9	114.9	114.9	114.9	114.9
N_1	0.64929	0.57415	0.55472	0.64845	0.6739	0.72856
M_1	0.91187	0.97602	1.02611	1.03428	1.00153	0.94422
$\log(A_2)$	13.08192	13.02887	12.80204	12.77982	12.8267	12.6187
E_2	98.9	98.9	98.9	98.9	98.9	98.9
N_2	1.6848	1.92951	0.95247	0.99978	1.67649	0.91314
M_2	0.94458	0.98068	0.83802	0.83637	0.86688	0.67518
$\log(A_3)$	15.19651	15.53805	15.61423	15.47713	15.42557	15.43836
E_3	114.9	114.9	114.9	114.9	114.9	114.9
N_3	9.61E-02	0.53267	0.46286	0.54003	0.59236	0.77517
M_3	0.35289	0.73919	0.77259	0.64713	0.60521	0.62791
$\Delta H_1/\Delta H$	0.53311	0.40634	0.2221	0.22451	0.24107	0.19933
$\Delta H_2/\Delta H$	0.37859	0.34664	0.32895	0.31056	0.26705	0.28582
ΔH	41.5032	55.28769	58.35707	66.58177	64.14673	69.20402
r	0.995812	0.988324	0.994159	0.999481	0.999742	0.999872

250 – 300 µm						
q^+	0.5	1	2	5	10	20
log(A_1)	16.55688	16.62673	16.70525	16.57555	16.4093	16.29358
E_1	118.1	118.1	118.1	118.1	118.1	118.1
N_1	0.66318	0.63224	0.76393	0.65537	0.96231	0.84582
M_1	0.96465	1.0244	1.0636	0.99175	0.95104	0.91974
log(A_2)	14.29303	13.73655	14.2598	13.86518	13.48142	13.45624
E_2	102.5	102.5	102.5	102.5	102.5	102.5
N_2	1.35173	1.08031	2.61096	1.00295	0.83764	0.9829
M_2	1.13652	0.97191	1.15918	0.97879	0.82156	0.80632
log(A_3)	16.21793	16.22497	16.28653	16.15718	16.08023	15.78885
E_3	118.1	118.1	118.1	118.1	118.1	118.1
N_3	0.56272	0.60701	0.77217	0.71259	1.05349	0.71633
M_3	0.80268	0.83076	0.83098	0.71569	0.61357	0.48534
$\Delta H_1/\Delta H$	0.35607	0.2899	0.31282	0.29095	0.3729	0.29065
$\Delta H_2/\Delta H$	0.17943	0.27634	0.31902	0.27345	0.25972	0.2761
ΔH	43.96593	52.27706	66.69253	63.1152	63.56093	68.62673
r	0.999965	0.999548	0.984491	0.999654	0.999959	0.999965

300 – 500 µm						
q^+	0.5	1	2	5	10	20
$\log(A_1)$	15.61577	15.64946	15.54124	15.42779	15.52643	15.40945
E_1	113.3	113.3	113.3	113.3	113.3	113.3
N_1	0.80745	0.7291	0.72166	0.62472	0.74299	0.8515
M_1	0.93496	0.93803	0.89804	0.83312	0.92286	0.90035
$\log(A_2)$	14.08228	13.39364	13.61231	13.34792	13.10659	13.17014
E_2	100.8	100.8	100.8	100.8	100.8	100.8
N_2	0.70373	1.07471	1.56803	0.92867	0.87385	1.1558
M_2	1.19906	0.96875	1.03623	0.91045	0.7779	0.86057
$\log(A_3)$	15.01724	15.4031	15.46926	15.29052	15.2079	15.01982
E_3	113.3	113.3	113.3	113.3	113.3	113.3
N_3	0.61782	1.64111	0.44423	0.86264	0.84911	0.82381
M_3	0.61814	0.74667	0.69827	0.68579	0.57208	0.54354
$\Delta H_1/\Delta H$	0.5233	0.57361	0.63701	0.31718	0.35316	0.26427
$\Delta H_2/\Delta H$	4.94E-02	0.24731	0.3217	0.2635	0.28645	0.25082
ΔH	47.38628	48.64121	61.95808	63.34755	68.94937	68.20955
r	0.999617	0.998836	0.989339	0.999866	0.999914	0.999977

Figure S2– *graphical representation of the CR and CHR cyclic temperature programs (temperature program in the left graph and the corresponding derivative relaxation response in the right graph).*

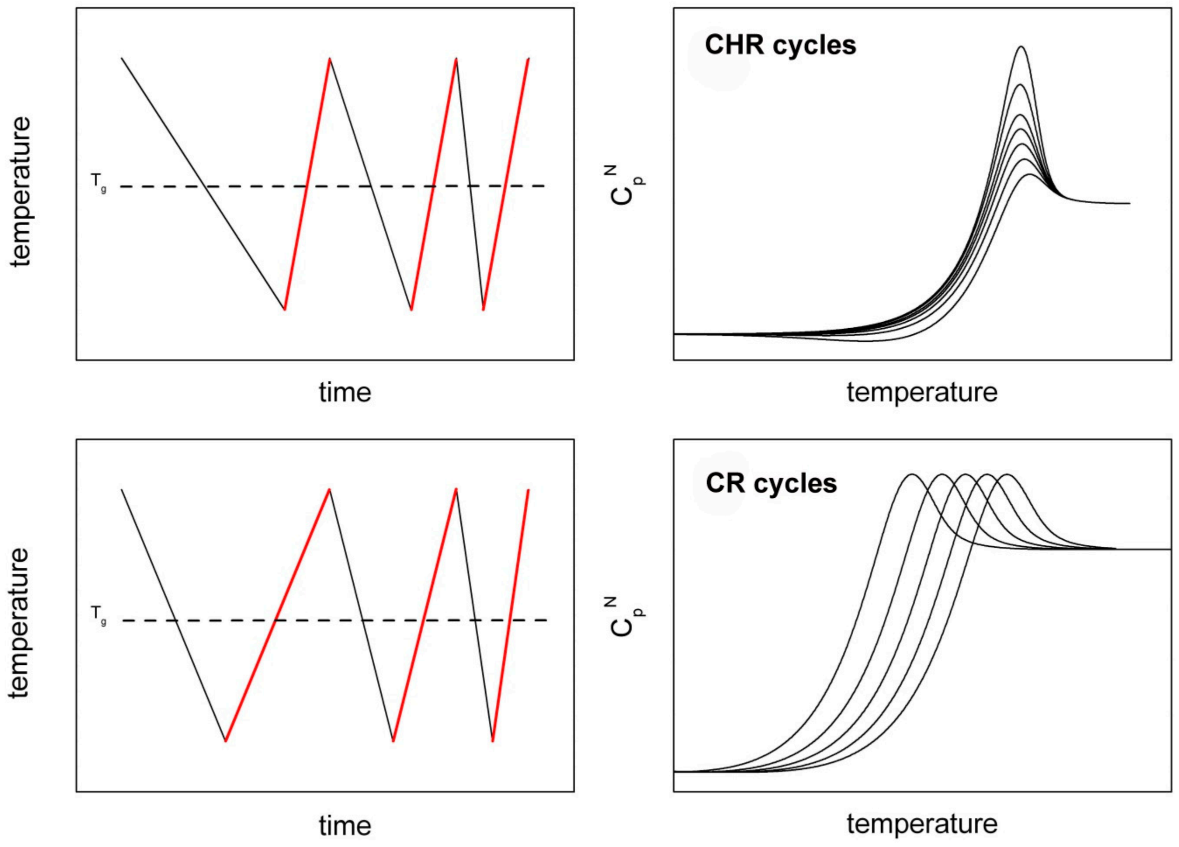

Figure S3– *optical micrographs corresponding to the as-prepared (left columns) and DSC-crystalized (right columns) NIF powders. Identical scale bar as shown for the as-prepared 125 – 180 µm powder is valid for all other micrographs. The legends inserted into micrographs correspond to the powder size in µm.*

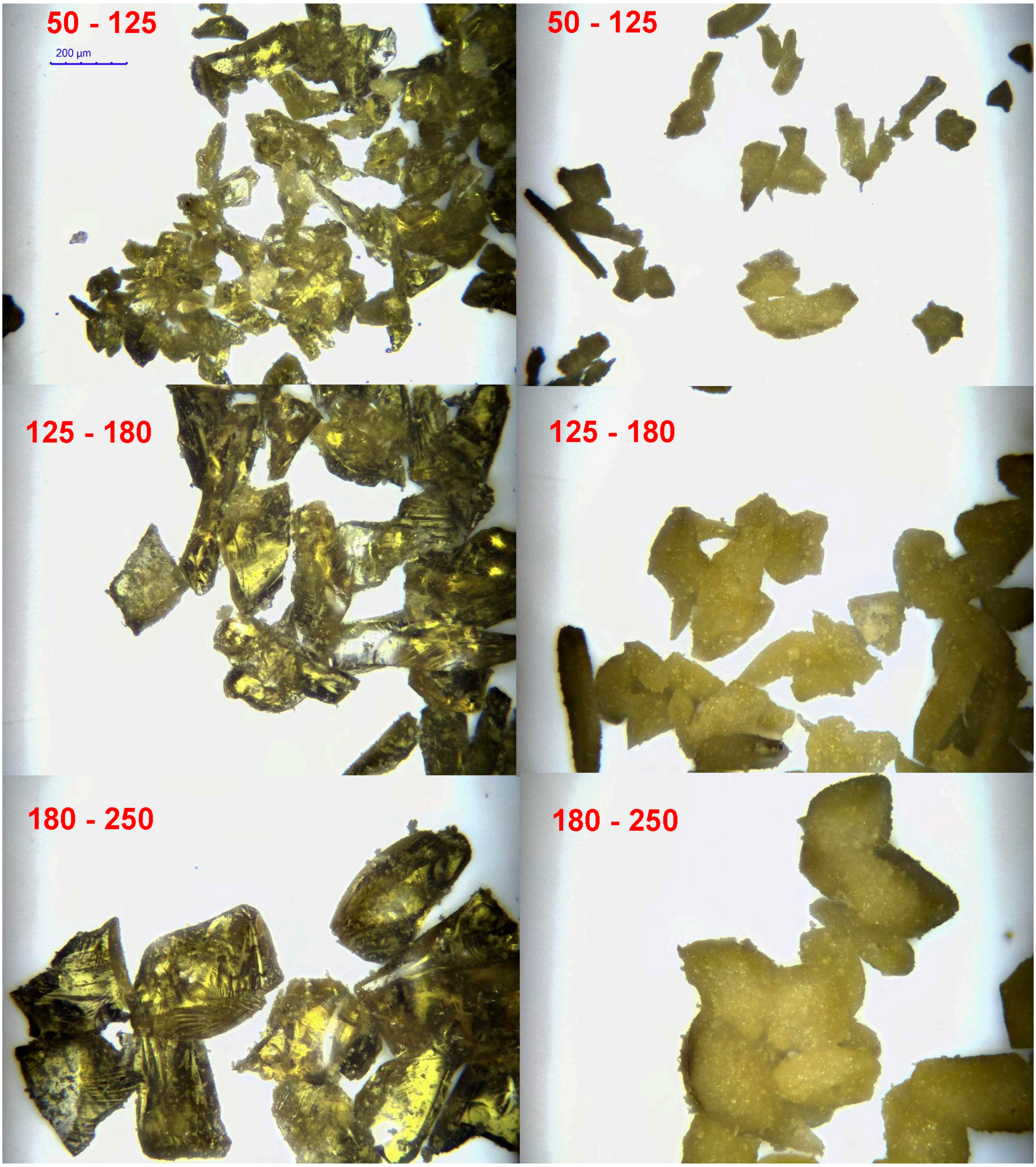

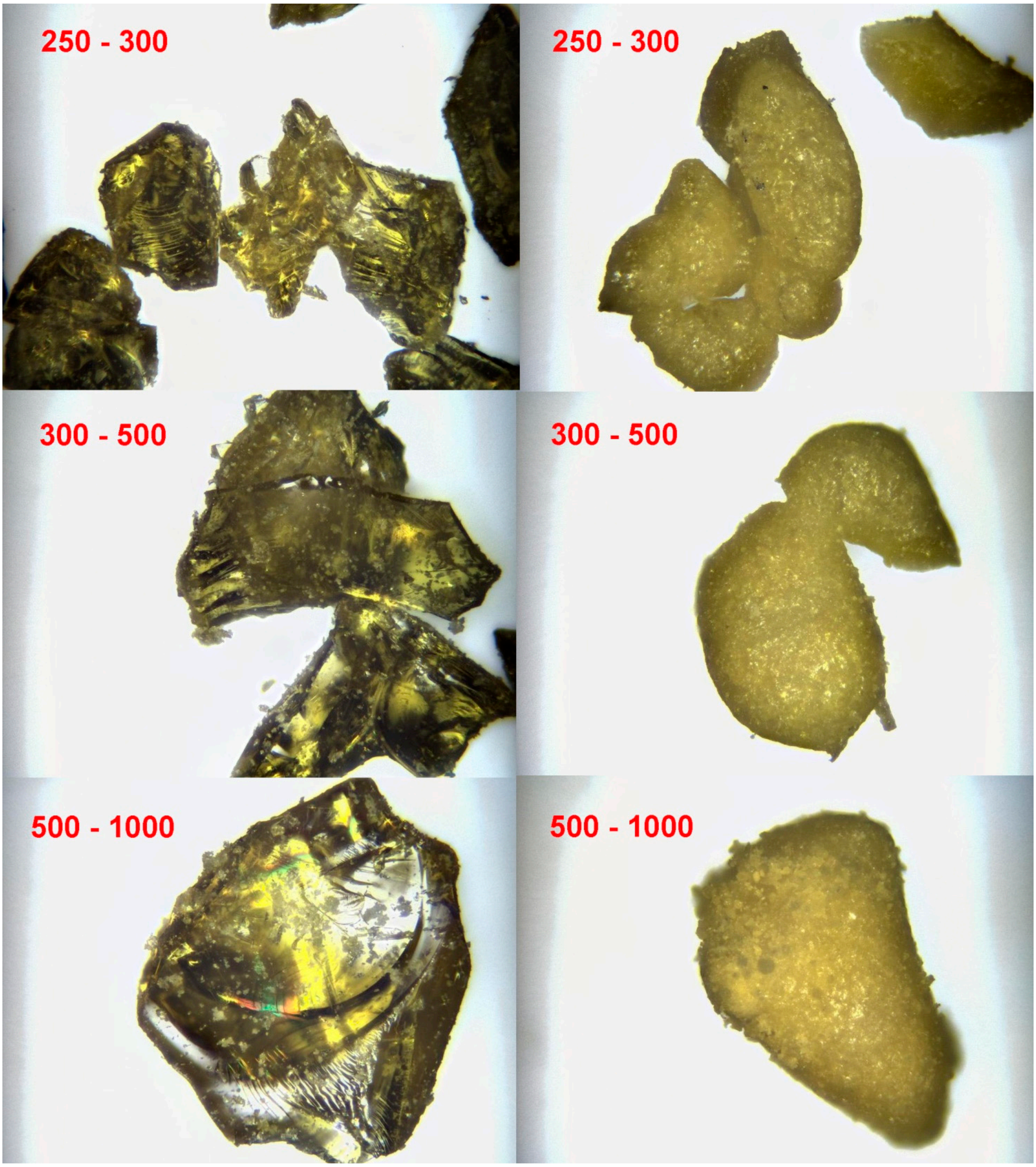
250 - 300
250 - 300
300 - 500
300 - 500
500 - 1000
500 - 1000

Figure S4– *alternative depictions of crystallization and melting enthalpies in J·g^{-1} (variants of Figs. 3E, 3F, 7C.*

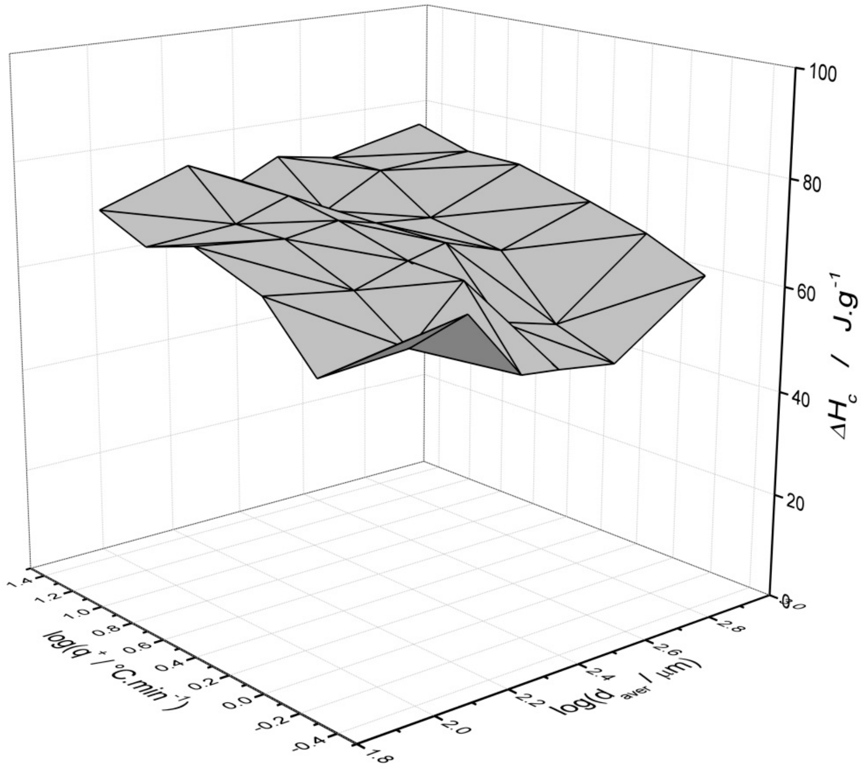

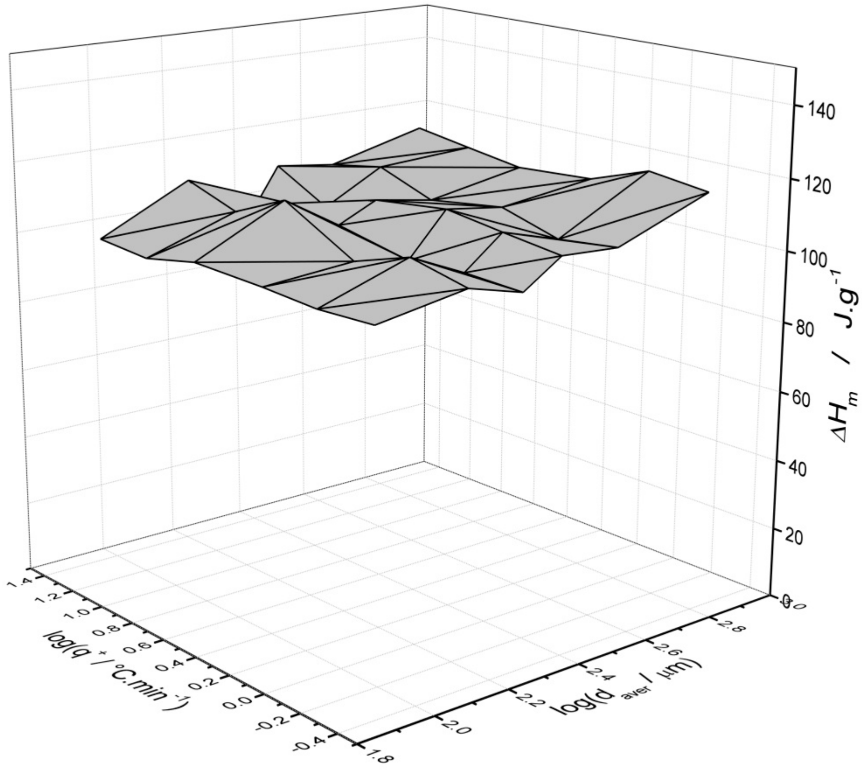

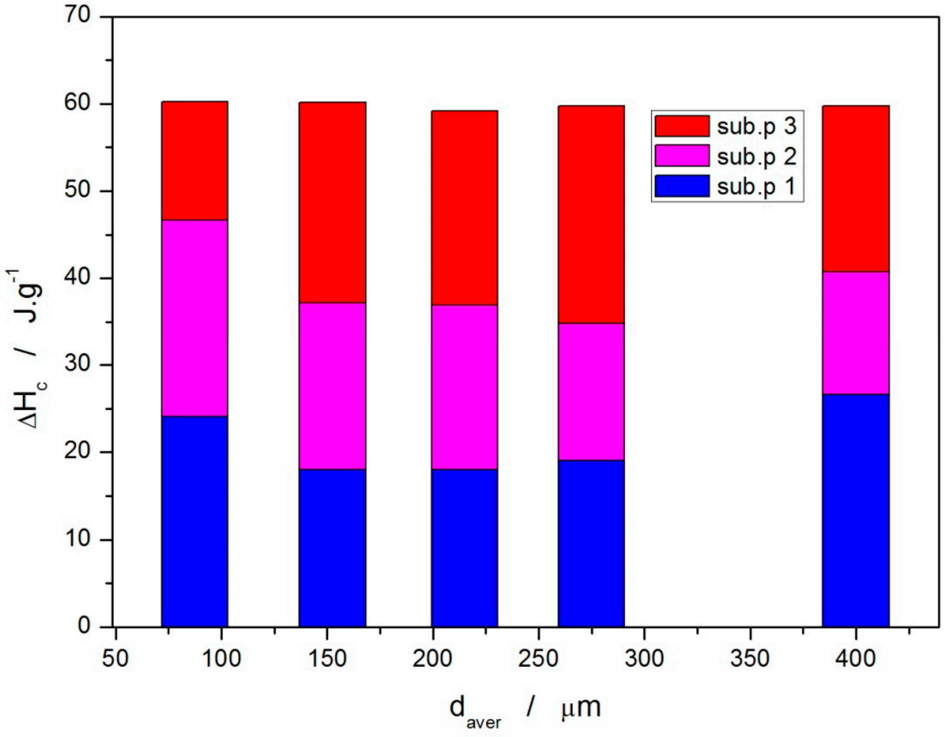

70
60
50
40
30
20
10
0
ΔH_c / J.g^-1
50
100
150
200
250
300
350
400
d_aver / μm
sub.p 3
sub.p 2
sub.p 1
